# Phylogenomic and phenotypic profiling of carbapenem-resistant Pseudomonas aeruginosa clinical isolates reveals lineage-specific resistance mechanisms and adaptive responses

**DOI:** 10.1099/mgen.0.001639

**Published:** 2026-02-09

**Authors:** Humberto Torres-Rodríguez, Elvira Garza-González, Claudia Adriana Colín-Castro, María Guadalupe Martínez-Zavaleta, Fabian Rojas-Larios, María del Rosario Vázquez-Larios, Christian Daniel Mireles-Dávalos, Daniel Romero-Romero, Pablo Hernan Sandoval-Villaseñor, Bernardo Alfonso Martínez-Guerra, Marlen Flores-Huacuja, Carlos Córdova-Fletes, Griselda García-Morales, Juan de Dios Castañeda-Duarte, Aldo Rafael Silva-Gamiño, César Adame-Álvarez, Brenda Berenice Ávila-Cárdenas, Cecilia Teresita Morales-De-La-Peña, Lourdes Hernández-Vicente, Frynne Magaly Flores-Castillo, Ibis De-la-Cruz-Hernández, Elena Victoria Choy-Chang, Juan Pablo Mena-Ramírez, Eduardo López-Gutiérrez, Mariana Gil-Veloz, Talia Pérez-Vicelis, Laura Isabel López-Moreno, Juan Manuel Barajas-Magallón, Paulina Fabiola González-Melgoza, Martha Irene Moreno-Méndez, Mario Galindo-Méndez, Eloísa Ramírez-Alanís, Ulises Garza-Ramos, Luis Esau López-Jácome

**Affiliations:** 1 Departamento de Bioquímica y Medicina Molecular, Facultad de Medicina, Universidad Autónoma de Nuevo León, Monterrey, Mexico; 2 Laboratorio de Microbiología Clínica, Instituto Nacional de Rehabilitación Luis Guillermo Ibarra Ibarra, Mexico City, Mexico; 3 Facultad de Medicina, Universidad de Colima, Colima, Mexico; 4 Instituto Nacional de Cardiología Ignacio Chávez, Mexico City, Mexico; 5 Instituto Nacional de Enfermedades Respiratorias “Ismael Cosío Villegas”, Mexico City, Mexico; 6 Diagnóstico Médico Integral Pasteur, Mexico City, Mexico; 7 Instituto Nacional de Ciencias Médicas y Nutrición Salvador Zubirán, Mexico City, Mexico; 8 Centro Nacional de Referencia de Inocuidad y Bioseguridad Agroalimentaria, del SENASICA, Mexico City, Mexico; 9 Hospital de Especialidades Pediátricas IMSS-Bienestar, Mexico City, Mexico; 10 Centro Médico Dr. Ignacio Chávez, ISSSTESON, Hermosill, Mexico; 11 Hospital Ángeles Morelia, Morelia, Mexico; 12 Hospital Centenario Miguel Hidalgo, Mexico City, Mexico; 13 Hospital Civil de Guadalajara “Fray Antonio Alcalde”, Guadalajara, Mexico; 14 Hospital General Juan María Salvatierra, Morelia, Mexico; 15 Hospital General “Dr. Raymundo Abarca Alarcón”, Acapulco, Mexico; 16 Hospital General “Dr. Miguel Silva”, Acapulco, Mexico; 17 Hospital General de Zona #46 IMSS, Mexico City, Mexico; 18 Hospital General de ZONA No.1 IMSS Nueva Frontera, Villahermosa, Mexico; 19 Hospital General de Zona No.21 IMSS Tepatitlán de Morelos, Tepatitlán de Morelos, Mexico; 20 Hospital Regional de Alta Especialidad de Oaxaca, Oaxaca City, Mexico; 21 Hospital Regional de Alta Especialidad del Bajío, León, Mexico; 22 Hospital Regional Tipo B de Alta Especialidad Bicentenario de la Independencia, León, Mexico; 23 Laboratorio Clínico Hospital Galenia, Cancún, Mexico; 24 Laboratorio Dipromi, Mérida, Mexico; 25 Laboratorio de Análisis Clínicos y Microbiológicos Futura Medica, Mexico City, Mexico; 26 Laboratorios del Centro, Mexico City, Mexico; 27 Laboratorios Galindo, Guadalajara, Mexico; 28 Sanatorio la Luz, Mexico City, Mexico; 29 Instituto Nacional de Salud Pública, Centro de Investigación sobre Enfermedades Infecciosas, Cuernavaca, Mexico; 30 Departamento de Biología, Facultad de Química, Universidad Nacional Autónoma de México, Mexico City, Mexico

**Keywords:** *Pseudomonas aeruginosa*, carbapenem resistance, oprD, efflux pumps, phylogenomics, genotype–phenotype association

## Abstract

Carbapenem resistance in *Pseudomonas aeruginosa* is a growing public health concern. Multiple mechanisms of antimicrobial resistance have been described. While surveillance often focuses on carbapenemase detection, non-carbapenemase mechanisms and their interplay with the genomic background remain underexplored. This study aimed to characterize how genomic background influences carbapenem resistance mechanisms and adaptive responses in carbapenem-resistant *P. aeruginosa* (CRPA). A total of 136 CRPA clinical isolates collected from 28 healthcare centres across Mexico were analysed through core genome phylogeny, sequence type (ST) assignment, resistome profiling, *oprD* variant analysis, bacterial growth kinetics under imipenem and meropenem exposure and qRT-PCR-based expression of *oprD*, *mexA*, *mexC*, *mexE* and *mexY* genes. Isolates clustered into PAO1 and PA14 phylogroups. ST309 was the most prevalent ST (29/136). *Pseudomonas paraeruginosa* lineage was also identified within these isolates. Phylogenetic clustering of antibiotic resistance genes was observed across phylogroups. In 36% (49/146) of the isolates, *β*-lactamases (*bla*_VIM_ 16%, *bla*_GES_11% and *bla*_IMP_ 11%) were identified with carbapenemase activity previously reported. Potentially inactivating *oprD* variants were observed in 75% (102/136) of isolates, with nonsense and frameshift variants associated with resistance phenotypes. Isolates harbouring carbapenemase-encoding genes (CEGs) exhibited stable lag phases regardless of antibiotic exposure, whereas isolates lacking CEGs showed significantly prolonged lag phases. Overexpression of *mexA, mexC* and *mexY* genes was observed in 39% (7/18), 17% (3/18) and 39% (7/18) of isolates, respectively, under antibiotic-free condition, and increased under carbapenem exposure. *mexA* expression was significantly higher in PAO1 isolates (6/18) under antibiotic-free condition and in PA14 isolates under imipenem exposure (5/18). Carbapenem resistance in *P. aeruginosa* is shaped by both phylogenetic background and antibiotic-driven stress responses. This study provides an integrated analysis of resistance mechanisms – including gene expression and physiological adaptation – across major phylogenetic lineages in clinical isolates recovered in Mexico, underscoring the importance of considering non-carbapenemase resistance pathways in surveillance and treatment strategies.

**Impact Statement**

Carbapenem-resistant *Pseudomonas aeruginosa* is a serious global health threat, yet surveillance has largely focused on carbapenemase detection. By combining genomic sequencing, phenotypic assays and gene expression profiling, this study shows that high-risk clones circulate widely in Mexico, with many resistant isolates lacking carbapenemase genes and relying on mechanisms such as OprD disruption and efflux pump presence. Unlike similar phylogenomic studies, this work includes phenotypic and expression data, linking resistance mechanisms to bacterial lineage and physiological responses and revealing how phylogeny shapes adaptation to antibiotic pressure. These results emphasized the need for integrated genomic and phenotypic surveillance strategies to detect emerging non-carbapenemase-mediated resistance in *P. aeruginosa*.

## Data Summary

The raw sequencing data have been deposited in the NCBI Sequence Read Archive (SRA) under the accession number PRJNA1211528. Individual accession numbers corresponding to each genome sequenced in this study are listed in Table S1 (available in the online Supplementary Material).

## Introduction

*Pseudomonas aeruginosa* is an opportunistic pathogen that can cause healthcare-associated infections, especially in immunocompromised individuals [1]. Although not intrinsically resistant to carbapenems, *P. aeruginosa* can acquire resistance to this class of antibiotics, which are often used as first-line treatment due to their broad-spectrum activity [2,3]. In consequence, the World Health Organization (WHO) has positioned carbapenem-resistant *P. aeruginosa* (CRPA) among the high-priority pathogens for research and development of new antimicrobial strategies [4].

In *P. aeruginosa*, high-level carbapenem resistance is often caused by the presence of carbapenemase-encoding genes (CEGs), the most common being the *bla*_VIM_, *bla*_IMP_ and, to a lesser extent, *bla*_GES_ variants [5,6]. However, in the absence of CEGs, resistance to carbapenems in *P. aeruginosa* is often attributed to the loss or inactivation of the OprD porin, which limits their uptake, as well as the overexpression of multidrug efflux pumps such as MexAB-OprM, MexCD-OprJ, MexEF-OprN and MexXY. These intrinsic mechanisms contribute variably to carbapenem resistance, and their regulation can be influenced by environmental cues and antibiotic exposure [7,12].

Resistance mechanisms in CRPA have been widely reported across diverse geographic regions, with prevalence and relative contribution often shaped by local epidemiological and genomic contexts [13]. For example, while carbapenem resistance in the USA is frequently linked to OprD variants, in Asia, a higher prevalence of CEGs, such as *bla*_VIM_ and *bla*_IMP_, has been reported [14,17]. The distribution and contribution of these mechanisms by region are often linked to a phylogenetic background of circulating strains [18,19]. Phylogenetic analyses have identified three major clades within *P. aeruginosa* populations spread worldwide – PAO1-like, PA14-like and the formerly referred PA7-like – which differ in virulence, resistance profiles and accessory genome content [18,20,22].

In Mexico, most studies have centred on the detection of CEGs in CRPA isolates [23,27]. However, a comprehensive understanding of non-carbapenemase resistance mechanisms remains limited [27]. Also, the distribution of major phylogenetic lineages in clinical settings across the country, and their contribution to the expression of resistance determinants, is yet to be explored [28]. Therefore, this study aimed to investigate the genomic and phenotypic determinants of carbapenem resistance in *P. aeruginosa* clinical isolates collected from multiple healthcare centres across Mexico. By integrating core-genome phylogenetic analyses, resistome profiling, variant analysis of *oprD*, efflux pump gene expression quantification and bacterial growth kinetics under carbapenem exposure, this study sought to characterize how genomic background influences carbapenem resistance mechanisms and adaptive responses.

## Methods

### Clinical isolates

A total of 136 *P*. *aeruginosa* clinical isolates collected between 2021 and 2023 from 28 centres in 16 Mexican states were included (Table 1). All centres belong to the INVIFAR network (Red Temática de Investigación y Vigilancia de la Farmacorresistencia in Spanish). Clinical isolates were recovered mainly from blood (35%), followed by bronchial samples (17%), urine (13%) and others (35%). All isolates were stored at −70 °C in nutrient broth with 15% glycerol in the strain collection until their use.

**Table 1.** Characteristics of participating centres

	Pu/Pr	No beds	ICU beds	State
**Hospitalization centres**				
Hospital Civil de Guadalajara ‘Fray Antonio Alcalde’	Pu	747	100	Jalisco
Hospital General ‘Dr. Miguel Silva’	Pu	443	16	Michoacan
Instituto Nacional de Rehabilitación ‘Luis Guillermo Ibarra Ibarra’	Pu	245	20	Mexico City
Instituto Nacional de Ciencias Médicas y Nutrición ‘Salvador Zubirán’	Pu	212	22	Mexico City
Hospital Regional Tipo B de Alta Especialidad Bicentenario de la Independencia	Pu	196	7	Mexico State
Hospital General de Zona #46 IMSS	Pu	190	18	Tabasco
Hospital Regional de Alta Especialidad del Bajío	Pu	184	16	Guanajuato
Hospital General de ZONA No.1 IMSS Nueva Frontera	Pu	180	28	Chiapas
Centro Médico Dr. Ignacio Chávez, ISSSTESON	Pu	174	4	Sonora
Instituto Nacional de Enfermedades Respiratorias ‘Ismael Cosío Villegas’	Pu	161	15	Mexico City
Hospital Centenario ‘Miguel Hidalgo’	Pu	144	17	Aguascalientes
Hospital de Especialidades Pediátricas IMSS-Bienestar	Pu	90	24	Chiapas
Hospital General de Zona No.21 IMSS Tepatitlán de Morelos	Pu	86	9	Jalisco
Hospital Regional de Alta Especialidad de Oaxaca	Pu	66	11	Oaxaca
Hospital Ángeles Morelia	Pr	55	12	Michoacan
Laboratorio Clínico Hospital Galenia	Pu	46	9	Quintana Roo
Sanatorio la Luz	Pr	28	3	Michoacan
Hospital General ‘Juan María Salvatierra’	Pu	157	18	Baja California Sur
Hospital General ‘Dr. Raymundo Abarca Alarcón’	Pu	227	7	Guerrero
Instituto Nacional de Cardiología ‘Ignacio Chávez’	Pu	208	30	Mexico City
**External laboratories**				
Laboratorio Dipromi	Pr	nd	nd	Michoacan
Laboratorio De Análisis Clínicos y Microbiológicos Futura Medica	Pr	nd	nd	Michoacan
Laboratorios Galindo	Pr	nd	nd	Oaxaca
Diagnóstico Médico Integral Pasteur	Pr	nd	nd	Mexico State
Laboratorios del Centro	Pr	nd	nd	Michoacan

ICU, intensive care unit; nd, no data; Pr, private; Pu, public.

### Antibiotic susceptibility testing

For each isolate, the minimum inhibitory concentration (MIC) was determined using the VITEK® 2 system (BioMérieux SA, Marcy-l'Étoile, France) and interpreted according to guidelines of M100 35^th^ edition 2025, Clinical and Laboratory Standards Institute (CLSI) [29].

Antibiotics evaluated were piperacillin/tazobactam, ceftazidime, cefepime, ceftazidime/avibactam, ceftolozane/tazobactam, imipenem (IPM), meropenem (MEM), amikacin (AMK), tobramycin (TOB), ciprofloxacin (CIP), levofloxacin (LVX), aztreonam (ATM) and colistin (COL).

### Whole-genome sequencing

Genomic DNA was extracted using the phenol-chloroform method [30] from fresh colonies on MacConkey agar. DNA concentration was determined using a Qubit 4.0 fluorometer (Invitrogen, USA), and purity was assessed using a NanoDrop 2000 spectrophotometer (Thermo Fisher Scientific, Massachusetts, USA). Absorbance ratios A260/A280 and A260/A230 ranging from 1.7 to 2.1 and 1.8 to 2.2, respectively, were considered acceptable for further analysis. The whole-genome sequencing was performed using the Illumina NextSeq 550 platform.

### Genome assembly and bioinformatic analysis

Adapters were removed with Trim Galore v0.4.4_dev (https://github.com/FelixKrueger/TrimGalore). *De novo* genome assembly was performed using Unicycler v0.4.9b [31]. Assembly quality was verified using QUAST v5.2 [32] and weeSAM (https://github.com/centre-for-virus-research/weeSAM).

Genome annotation was performed using Prokka v1.14.6. Multilocus sequence typing (MLST) was assigned using the PubMLST tool (https://pubmlst.org), and pangenome analysis was conducted using Panaroo with a strict error cleaning mode and a core genome threshold of 99%. Reference sequences included PAO1 (RefSeq: GCF_000006765.1), PA14 (RefSeq: GCF_000014625.1) and *Pseudomonas paraeruginosa* PA7 (GCF_000017205.1).

Phylogenetic trees based on the core genome alignment and SNPs were constructed using IQ-TREE2 [33] with model selection (MFP+MERGE), 1,000 ultrafast bootstrap replicates and partitioned by gene. Visualization was done in iTOL. Variant calling was performed using Snippy v4.6.0 (https://github.com/tseemann/snippy), using PAO1 as a genome reference.

Average nucleotide identity (ANI) values were calculated and visualized with ANIclustermap (https://github.com/moshi4/ANIclustermap), and antimicrobial resistance genes (ARGs) were identified using the web-based software application EPISEQ® CS (bioMérieux, Marcy-l’Étoile, France). To minimize tool-specific bias and ensure accurate gene assignment, resistance gene detection was cross-validated using ABRicate (CARD and Resfinder databases) (https://github.com/tseemann/abricate). Concordant gene calls were considered for further analyses.

For the analysis of sequence variation in *oprD* and efflux pump-related genes, a phylogroup-specific reference-based approach was applied. For each isolate, gene sequences were compared against the corresponding reference strain according to its phylogenetic assignment: *P. aeruginosa* PAO1 for isolates clustering within the PAO1 phylogroup and *P. aeruginosa* PA14 for isolates belonging to the PA14 phylogroup. Nucleotide sequences were manually curated to ensure full-length gene representation, including those containing frameshift variants. Multiple sequence alignment was performed using MAFFT v7.526 [34], and a maximum likelihood phylogenetic tree was constructed with IQ-TREE2. To evaluate the impact of resistance mechanisms on carbapenem non-susceptible, association analysis was performed between the MIC values for IPM and MEM and the presence of CEGs, as well as the type of *oprD* gene variants (insertion sequence, nonsense, frameshift and missense variants).

### Growth kinetics

Growth kinetics were performed for selected *P. aeruginosa* isolates under three experimental conditions: (A) in the absence of antibiotic, (B) in the presence of IPM and (C) in the presence of MEM. The concentrations of IPM and MEM used corresponded to the respective MICs observed for each isolate.

Isolates were grown overnight on MacConkey agar at 37 °C; turbidity was adjusted to 0.5 McFarland and inoculated into Lysogeny broth in the three conditions (A, B and C) in 96-well plates.

Growth was monitored every 10 min at 600 nm over 48 h at 37 °C using a Cytation I image reader (BioTek, Vermont, USA), under continuous shaking. The exponential growth phase was used to determine RNA sampling timepoints for each strain and condition to be used in the expression analysis.

### RNA extraction and gene expression analysis

Total RNA was extracted from bacterial cultures grown under the same three experimental conditions previously described, using TRIzol LS (Thermo Fisher Scientific), followed by isopropanol precipitation and ethanol washing. RNA concentration and purity were assessed by NanoDrop 2000 (Thermo Fisher Scientific). DNase I treatment was performed to remove genomic DNA, and cDNA was synthesized using M-MLV Reverse Transcriptase kit (Thermo Fisher Scientific), following the manufacturer’s protocol.

Expression levels of *oprD*, *mexA*, *mexC*, *mexE* and *mexY* were quantified via qPCR, normalized to the endogenous reference gene *rpsL*. All reactions were performed in technical triplicate. Relative expression was calculated using the Livak 2^-ΔΔCt^ method, and results were expressed as log_2_FoldChange values compared to the reference strain *P. aeruginosa* PAO1. Genes with log_2_FoldChange ≤−1.0 were considered underexpressed, while those with log_2_FoldChange ≥1.0 were considered overexpressed. Primer sequences used are listed in Table S2 [35].

### Statistical analysis

A series of statistical analyses was performed to evaluate associations between gene expression, growth dynamics and resistance phenotypes. All statistical analyses were conducted using R software (https://www.r-project.org/, accessed 10 May 2025). A summary of the tests applied is provided in Table S3.

## Results

### Susceptibility profile

MIC ranges and MIC_50_ and MIC_90_ observed are presented in Table 2. Carbapenems showed the highest resistance rates (IPM and MEM, 90%), followed by fluoroquinolones (LVX and CIP, 100 and 71%, respectively) and aminoglycosides (TOB and AMK, 100 and 64%, respectively). The lowest resistance rate was observed to ATM (9%). All isolates exhibited an intermediate susceptibility profile to COL, consistent with current CLSI criteria, in which the susceptible category has been removed, and an MIC ≤2 mg l^−1^ is classified as intermediate. Complete results for the susceptibility tests are shown in Table S4.

**Table 2.** MIC ranges and MIC_50_ and MIC_90_ values of the studied isolates

Antibiotic	MIC range			MIC_50_	MIC_90_
Piperacillin/tazobactam	<4	–	>128	64	>128
Ceftazidime	1	–	>64	32	>64
Cefepime	1	–	>32	16	>32
Cefotaxime	0.5	–	>32	>32	>32
Ceftazidime/avibactam	1	–	>16	>16	>16
Imipenem	2	–	>16	>16	>16
Meropenem	<0.125	–	>16	>16	>16
Amikacin	1	–	>64	>64	>64
Tobramycin	6	–	33	6	24
Ciprofloxacin	0.006	–	>4	>4	>4
Levofloxacin	6	–	44	6	32
Aztreonam	6	–	43	21	31
Colistin	<2	–	<2	<2	<2

### Genome assembly

The draft genomes of the 136 *P*. *aeruginosa* clinical isolates had an average genome size of 6,775,960 bp, with a G+C content of 66 mol%. Assemblies resulted in an average of 91 contigs per genome. The N50 and L50 values were 285,542 bp and 10, respectively, indicating relatively contiguous assemblies. The average genome coverage was 95.83%, with an average sequencing depth of 69×. All sequences showed Phred quality scores >30 across the read length, ensuring high-confidence base calling for genome assembly. Quality parameters for each assembly are detailed in Table S5.

### Molecular epidemiology

Based on MLST analysis, a total of 34 distinct sequence types (STs) were identified among 127 *P*. *aeruginosa* clinical isolates included in this study, and for 9 clinical isolates, not previously reported STs were detected.

The most frequent STs observed were ST309 (29/136, 21%), followed by ST235 (14/136, 10%), ST111 (13/136, 10%) and ST2731 (11/136, 8%). The remaining STs were detected in fewer than ten isolates. Five novel STs were identified in eight isolates: ST5358 (1/136), ST5383 (2/136), ST5384 (2/136), ST5385 (2/136) and ST5386 (1/136). The allelic profiles of these novel STs were submitted to and validated by the PubMLST database. A detailed list of the clinical isolates and their corresponding STs is provided in Table S6.

### Similarity at the nucleotide level

ANI revealed three groups of isolates with high intra-group similarity (>99%). The two largest groups shared an average similarity of ~98%, while the smaller group showed less than 94% similarity to the other two (Fig. S1).

### Phylogenomic analysis

A total of 13,961 genes were identified and classified into four categories: core, soft-core, shell and cloud. Core genes (4,283) and soft-core genes (959) together accounted for 37.6% of the pangenome, while shell genes (1,858) and cloud genes (6,861) accounted for 62.5%.

In the maximum likelihood tree constructed from the core genome phylogeny, the alignment of the reference strains (*P. aeruginosa* PAO1, PA14 and *P. paraeruginosa* PA7, formerly referred to as *P. aeruginosa* PA7) with the analysed isolates confirmed the identity. The *P. paraeruginosa* reference strain forms a clade with three of the study isolates.

**Fig. 1.**
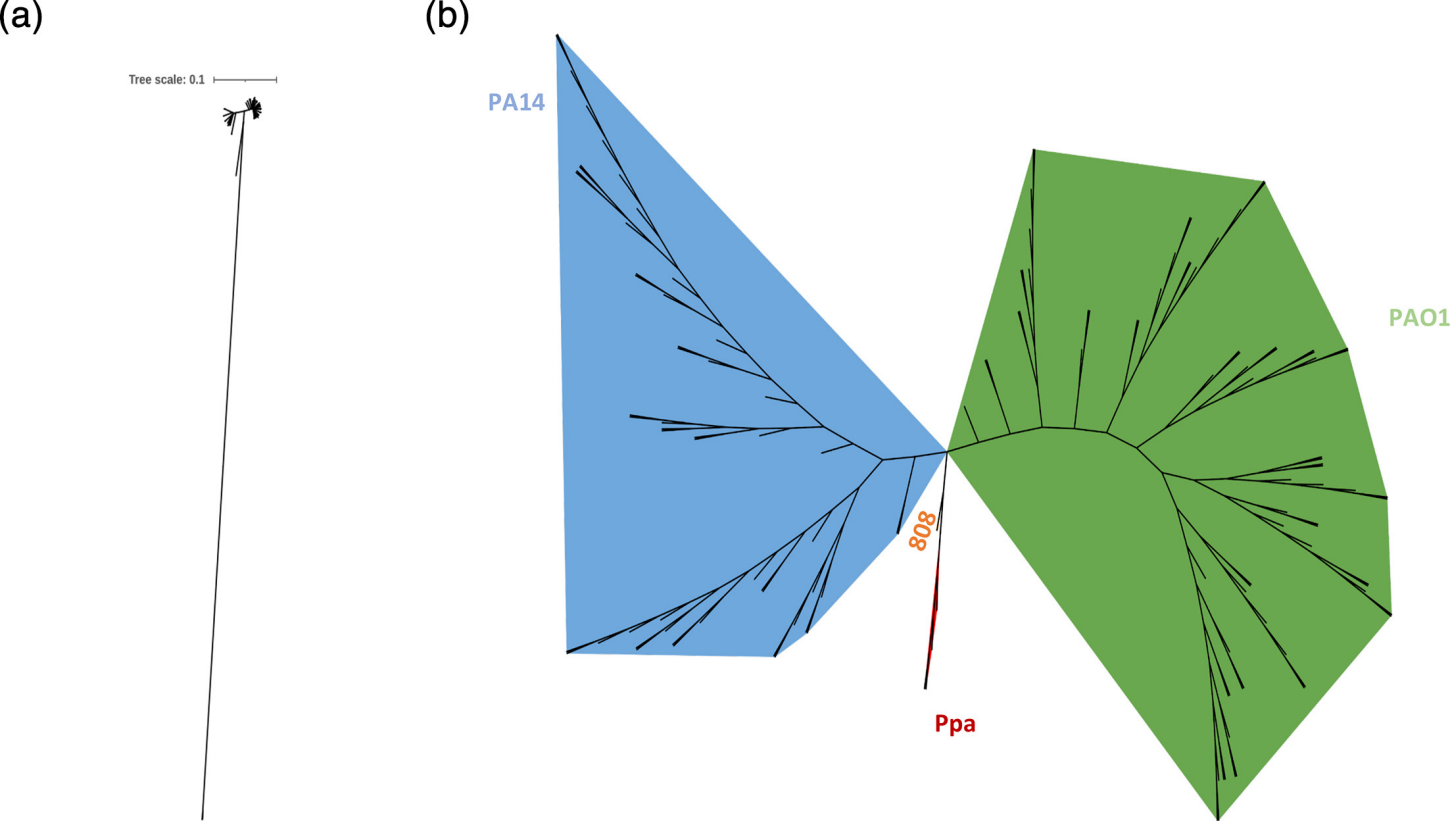
Unrooted phylogenetic tree based on the core genome of the 136 *P. aeruginosa* isolates included in this study. (a) Scaled tree illustrating the evolutionary distance of the *P. paraeruginosa* (Ppa) lineage relative to other isolates. (b) Unscaled representation to improve visualization of clustering patterns. Three main phylogenetic groups were identified, corresponding to reference strains PAO1 (green), PA14 (blue) and Ppa (red).

An unrooted tree enabled the visualization of the phylogenetic distribution of the analysed isolates (Fig. 1a). Based on tree topology and clustering patterns, three major groups are observed, corresponding to the typical lineages of *P. aeruginosa* represented by the reference strains and the *P. paraeruginosa* (Ppa) lineage. Additionally, an atypical strain was identified between the PA14 phylogroup and the Ppa lineage, which did not cluster within any of the three main clades (Fig. 1b). The PAO1 phylogroup included the largest number of isolates (79/136, 58%), followed by PA14 phylogroup (53/136, 40%), while Ppa lineage (3/136, 3%) was the least represented group (Fig. 1b).

When STs were mapped into the phylogenetic tree based on the core genome, the most frequent STs, such as ST309 and ST235, clustered within the PA14 phylogroup. In contrast, ST111 and ST2731 were the most representative within the PAO1 phylogroup. The Ppa lineage exhibited the novel STs ST5385 and ST3560.

**Fig. 2.**
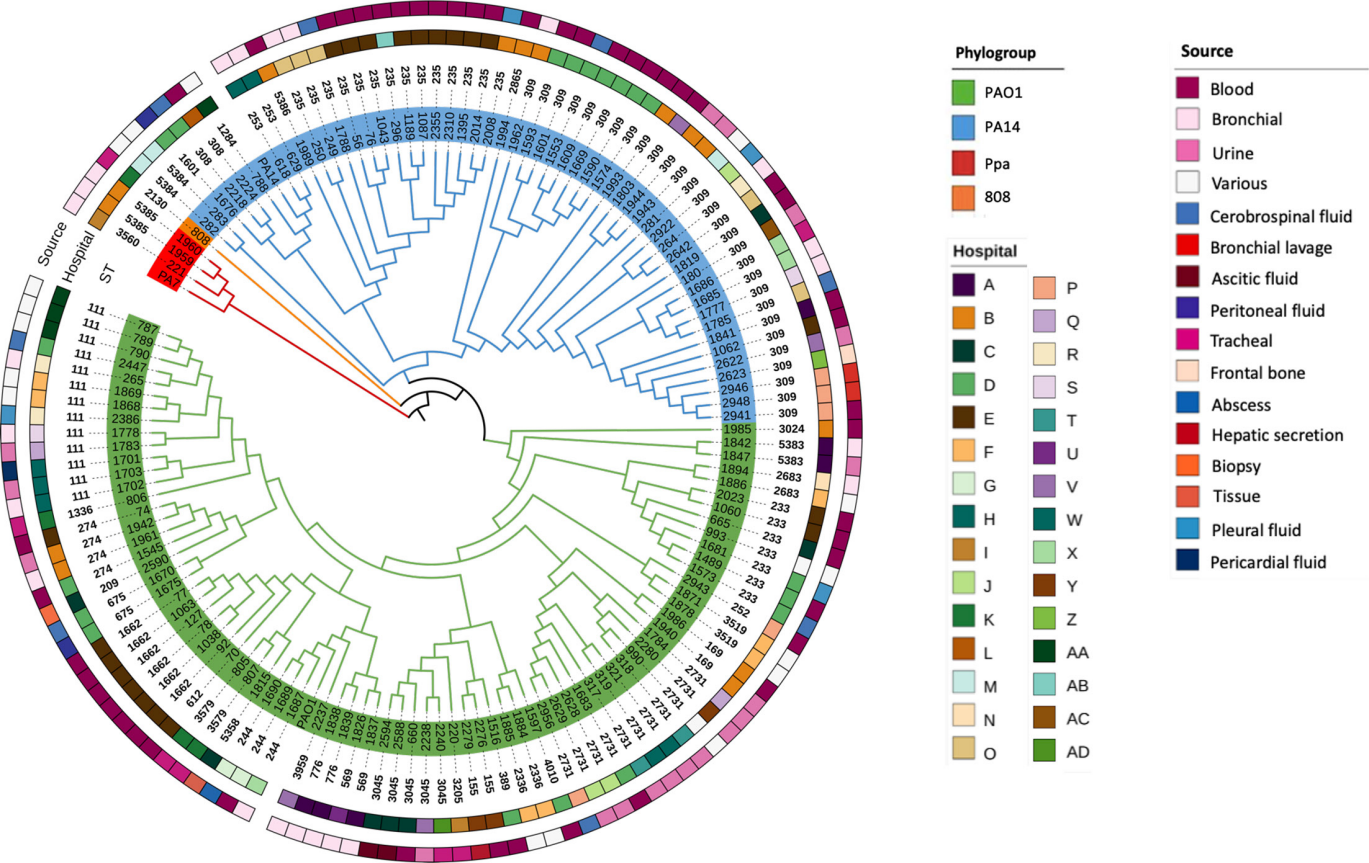
Maximum likelihood phylogenetic tree based on the core genome alignment of the analysed isolates and reference strains, annotated with ST (MLST), hospital of origin and clinical source. Branch lengths were ignored to a better visual display. Ppa, *P. paraeruginosa*.

Regarding hospital distribution, STs were dispersed across different hospitals, with no association between specific ST and a particular hospital, except for a few isolates, such as ST1662, which were isolated from the same institution (Fig. 2).

### Variant analysis

The maximum likelihood tree based on variant analysis preserved the previously identified phylogroups (PAO1 and PA14) and the Ppa lineage in the tree constructed from the core genome analysis.

**Fig. 3.**
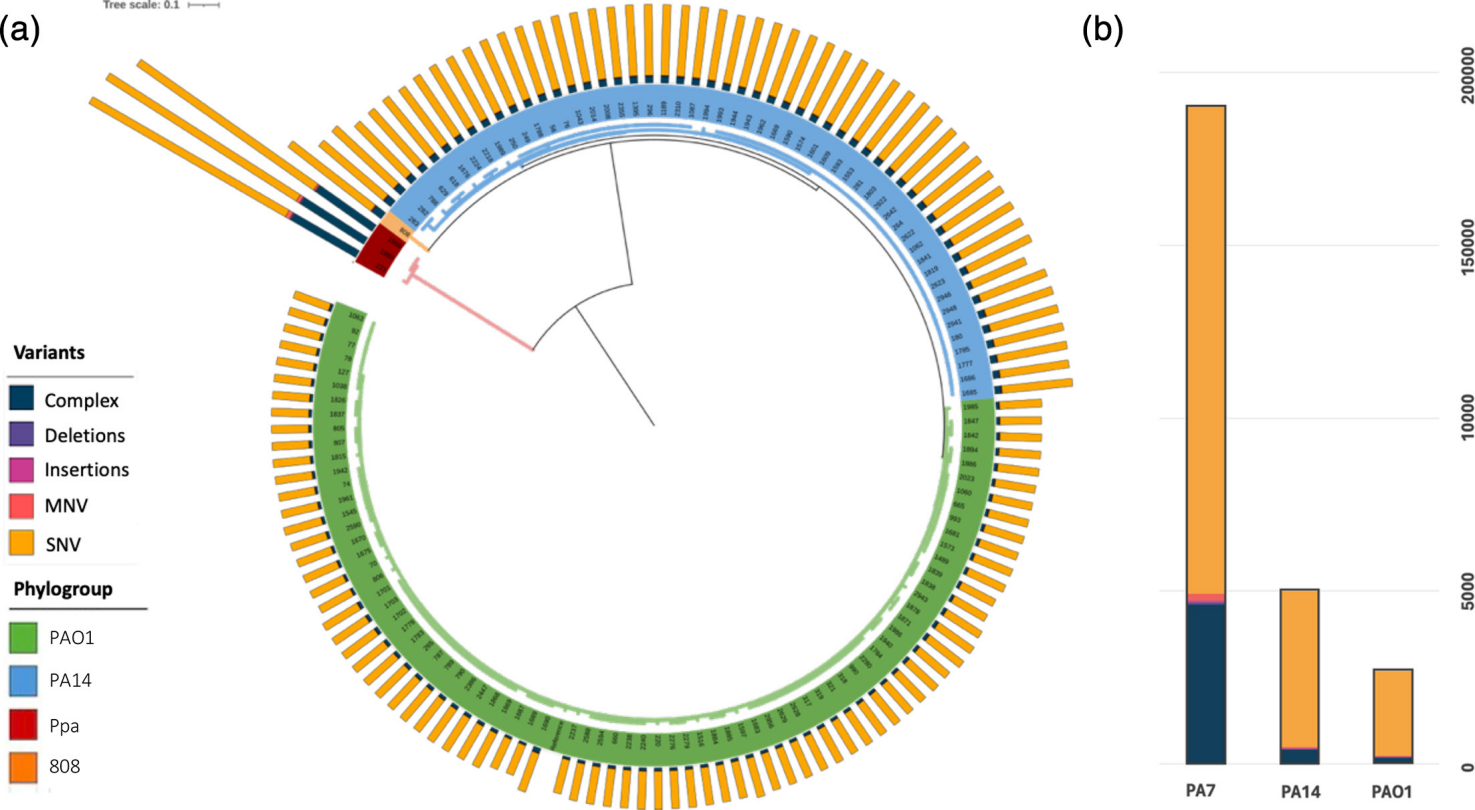
(a) Maximum likelihood phylogenetic tree based on variants. Outer ring represents the variant load for each individual isolate. (b) Number and type of variants in each phylogroup. SNV, single-nucleotide variant; MNV, multiple nucleotide variant; Ppa, *P. paraeruginosa*.

Examination of the total number of variants revealed a comparable accumulation of variants in isolates within the same phylogroup. The Ppa lineage exhibited the highest variant load compared to PAO1 and PA14 (Fig. 3a).

Regarding the type of variants, single-nucleotide variants were the most frequent across all three phylogroups. However, complex structural variants, such as genomic rearrangements, were more common in the Ppa lineage (Fig. 3b). Individual variants for each isolate are listed in Table S7.

### Resistome

**Fig. 4.**
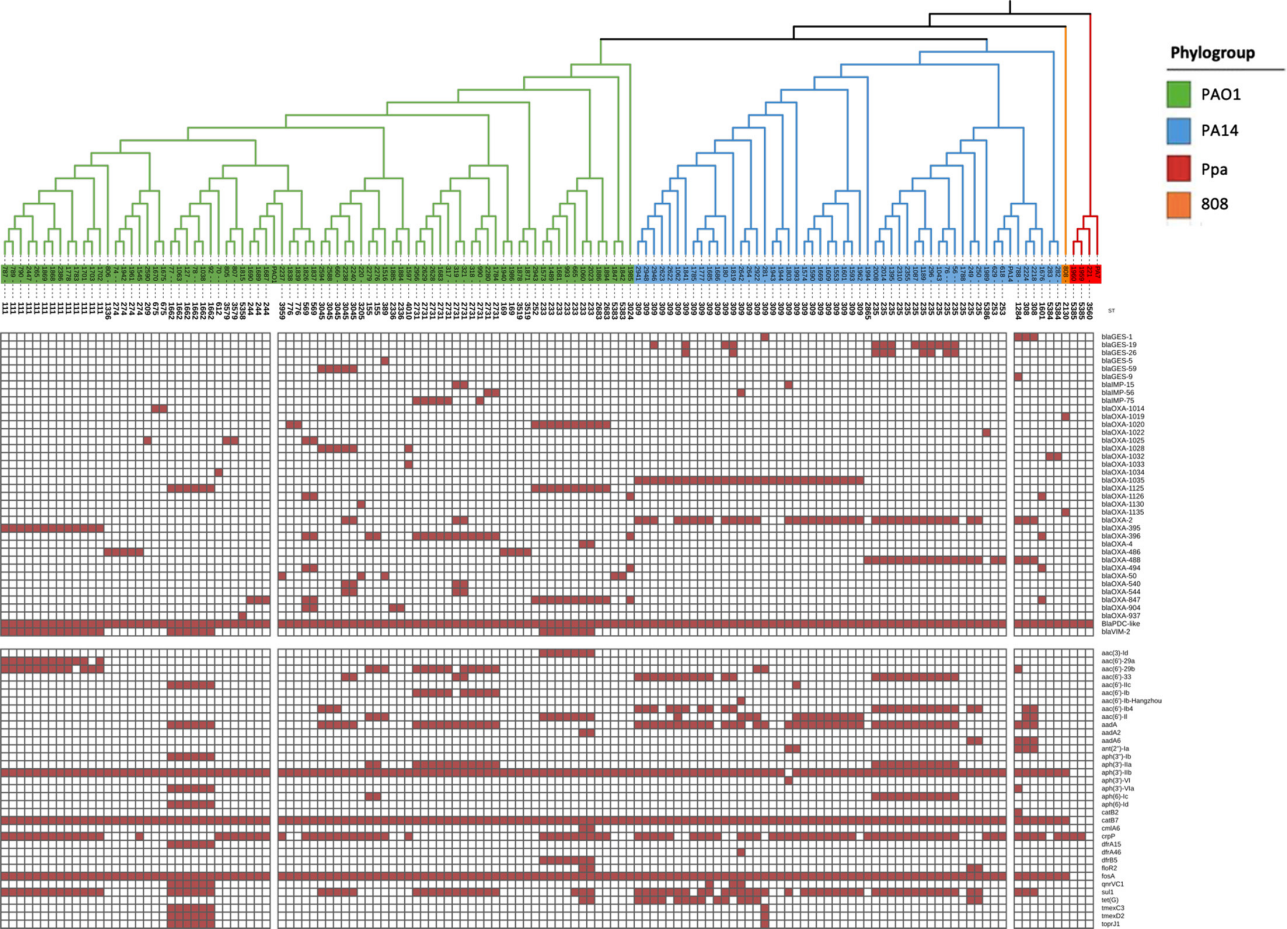
Presence/absence matrix of acquired resistance genes identified in the analysed *P. aeruginosa* isolates, grouped by phylogroup. Only genes with at least one copy present in any of the strains are shown. Phylogroups are represented in colour green (PAO1), blue (PA14) and red (Ppa, *P. paraeruginosa*).

A phylogenetic distribution of resistance genes was observed: *bla*_GES_ genes were clustered in the PA14 phylogroup, while *bla*_VIM_ genes were frequent in the PAO1 phylogroup. *bla*_IMP_ genes were distributed across both phylogroups, while *bla*_OXA_ was distributed along PAO1 and PA14 phylogroups. A total of 38% (*bla*_VIM_ 16%, *bla*_GES_ 11% and *bla*_IMP_ 11%) of the clinical isolates harboured *β*-lactamase genes with carbapenemase activity reported. In contrast, *bla*_OXA_ variants corresponded to allelic variants of *bla*_OXA-50_, an intrinsic chromosomally encoded *β*-lactamase of *P. aeruginosa*. These variants do not confer carbapenemase activity and were therefore not considered acquired carbapenem resistance determinants. No *bla*_OXA_ carbapenemases were detected in this dataset. Clinical isolates from the Ppa lineage did not carry any acquired *β*-lactamase genes (Fig. 4).

To visualize these differences in the resistance genes, a principal coordinate analysis was performed based on the presence/absence matrix of the resistance genes. In Fig. S2, the first two principal components account for 16.88% (PC1) and 11.01% (PC2) of the total variability. Isolates belonging to the PA14 phylogroup exhibit greater dispersion and are mostly clustered toward the right side of the plot. In contrast, PAO1 phylogroup isolates form a more compact cluster located on the left side. The Ppa lineage occupies an intermediate region of the plot with low dispersion, indicating reduced internal variability.

### *oprD* variant analysis

**Fig. 5.**
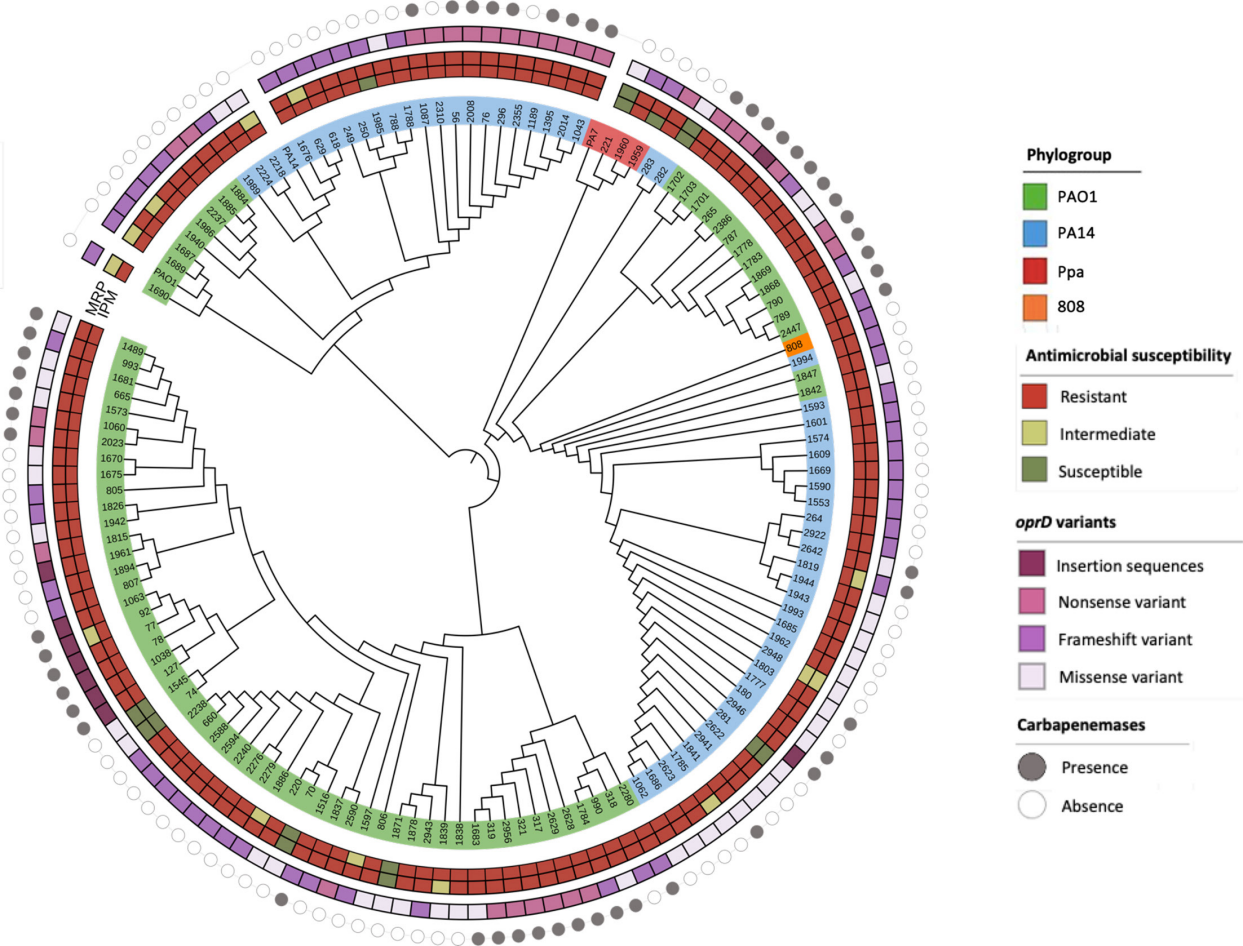
Cladogram of oprD gene sequences from the analysed *P. aeruginosa* isolates and reference strains (PAO1, PA14 and Ppa). Ppa, *P. paraeruginosa*.

Of the 136 clinical isolates analysed, 62% did not harbour genes encoding carbapenemases. To investigate alternative resistance mechanisms in the collection of isolates, a detailed variant analysis of the *oprD* gene was performed. A cladogram was constructed based on the *oprD* nucleotide sequences, revealing a phylogenetic structure previously defined through core genome and variant analyses (Fig. 5).

Notably, none of the isolates retained the wild-type *oprD* sequence, as all exhibited at least one missense variant. Furthermore, 75% (102/136) of the isolates carried variants predicted to disrupt OprD function, including insertion sequences, nonsense and frameshift variants. Insertions of transposons and phage-derived sequences in *oprD* were exclusively observed in isolates belonging to the PAO1 phylogroup.

When compared with antimicrobial susceptibility, a consistent pattern was observed between carbapenem resistance and the presence of a resistance mechanism. Isolates susceptible to both carbapenems lacked CEG and potentially disruptive *oprD* variants. In contrast, isolates resistant to both IPM and MEM exhibited at least one of these resistance mechanisms, or both.

A pattern between genotype and phenotype was observed. No statistical test was performed given carbapenem-susceptible isolates showed no variability in the analysed resistance determinants. In carbapenem-susceptible isolates to IPM and MEM, CEGs were absent, and only missense variants were observed in *oprD*. As MIC values increased, variants potentially disrupting OprD function – such as nonsense and frameshift variants – began to appear and were associated with intermediate susceptibility or low-level resistance phenotypes for IPM but remained susceptible or showed lower MICs for MEM. In contrast, elevated MICs (>16 µg ml^−1^) for both carbapenems were only observed in isolates carrying carbapenemase genes and potentially inactivating *oprD* variants (Fig. S3).

### Efflux pump presence

The presence of resistance–nodulation–division (RND) family efflux pumps was conserved across all analysed isolates. However, in three isolates belonging to the PAO1 phylogroup, the *mexEF-oprJ* and *mexXY* operons were not detected under the applied sequence identity and coverage thresholds (Fig. S3). Given the high conservation of these operons in *P. aeruginosa*, this likely reflects sequence divergence rather than true gene absence.

### Expression analysis and growth kinetics

A subset of 18 isolates was selected based on the diversity observed in the full strain set (*n*=136), including variation in phylogroup, CEG presence, *oprD* variant types and susceptibility profiles (Table S8). Growth kinetics and expression assays were performed to validate the correlation between genotypic features and resistance phenotypes.

**Fig. 6.**
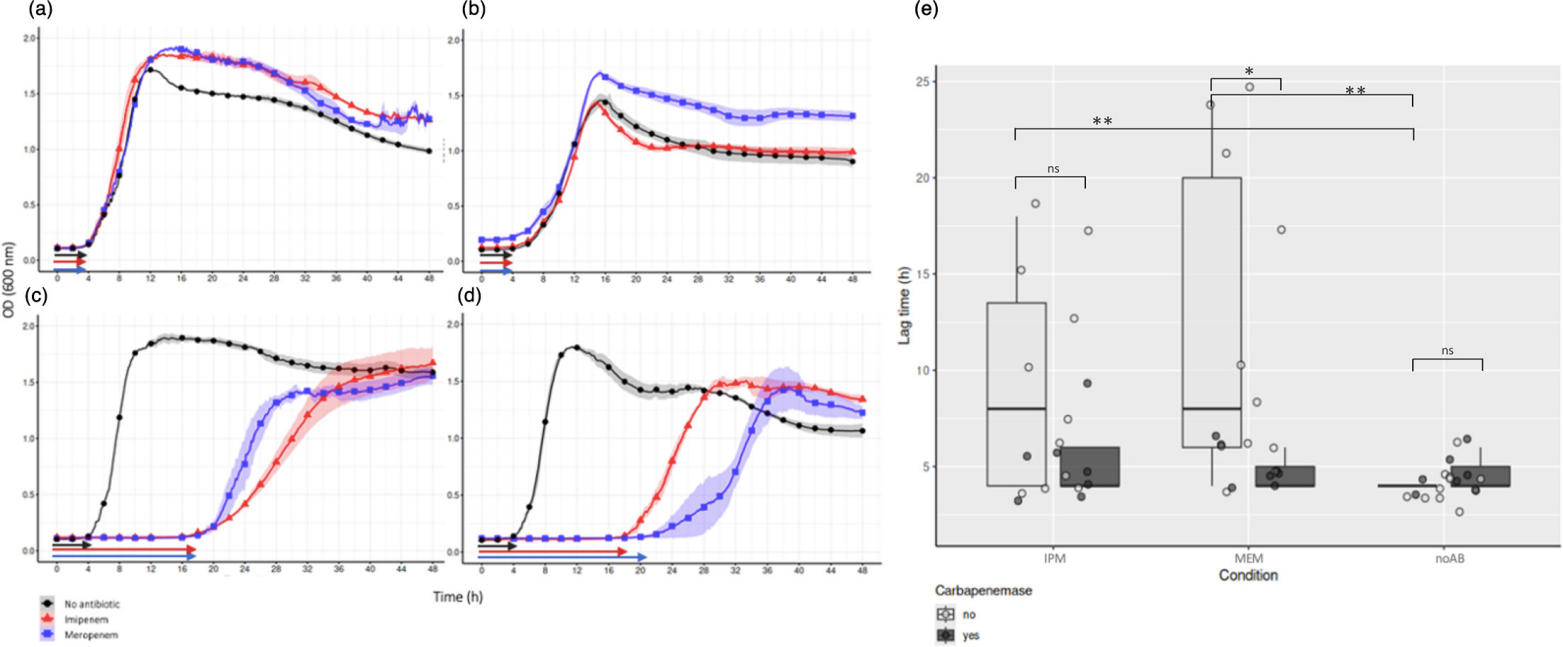
(a–d) Representative growth curves of four *P. aeruginosa* isolates with (**a, b**) or without CEGs (**c, d**) under three conditions [antibiotic-free (condition A), imipenem (condition B) and meropenem (condition C)]. Arrows indicate the estimated end of the lag phase for each isolate. Arrow colours correspond to lag phase and experimental conditions: black (no antibiotic), red (imipenem) and blue (meropenem). (e) Lag time of 18 *P. aeruginosa* isolates with or without CEGs under three conditions. IPM, imipenem; MEM, meropenem; noAB, no antibiotic; ns, no significance.

Growth curve analysis revealed phenotypic differences associated with CEG presence (Fig. 6a). Isolates harbouring these genes exhibited a shorter lag phase compared to those lacking CEGs. Based on these growth profiles, RNA extraction timepoints were determined during the exponential phase and adjusted individually for each strain and experimental condition. Complete growth curves and corresponding RNA sampling points for all isolates across the three previously specified conditions (A, B and C) are presented in Fig. S5.

Most isolates exhibited higher growth rates in condition A than in conditions B and C, particularly those lacking CEGs (Fig. 6). In contrast, isolates with CEGs showed comparable or even higher rates in conditions B and C than in condition A. Nevertheless, the overall growth rate did not differ significantly between CEG-positive and CEG-negative groups (*P*>0.05). Individual growth rate values for each clinical isolate across the three conditions A, B and C are provided in Table S9.

Differences in lag time were also observed (Fig. 6e). Among isolates with CEGs, lag time remained consistent across the three tested conditions (A, B and C). In contrast, isolates with non-CEGs exhibited a significantly prolonged lag phase under conditions B and C compared to condition A (**P=0.0022).

When the lag phase time between isolates with and without CEGs was compared within each condition, a statistically significant difference was found under MEM exposure (*P=0.014), while no significant differences were found under conditions A or B. Although not statistically significant, a trend toward increased lag time under IPM exposure was noted in non-CEG isolates. All individual lag time values are provided in Table S10.

#### Intrastrain gene expression under antibiotic-free condition

Gene expression was determined in the selected isolates under antibiotic-free condition (Fig. S9). All 18 selected isolates exhibited downregulation of *oprD*, while a subset showed overexpression of efflux pump genes: *mexA* and *mexY* were overexpressed in 39% of isolates (7/18) and *mexC* in 17%(3/18), while *mexE* was not overexpressed in any isolate. Detailed log_2_FoldChange values and their interpretation are available in Table S11.

No significant differences were observed between gene expression levels and carbapenem susceptibility phenotypes. However, the expression of *mexA* was significantly higher in isolates belonging to the PAO1 phylogroup compared to those in PA14 (*P*=0.0136; Fig. S7). For the remaining genes (*oprD*, *mexY*, *mexC* and *mexE*), expression levels did not differ significantly between phylogroups (Fig. S7).

Analysis of sequence variation in the *mexR* repressor gene revealed a phylogroup-dependent pattern (*P*=0.0086): PAO1 isolates harboured more diverse and potentially functional variants, whereas PA14 isolates carried only synonymous substitutions (Fig. S9).

A positive correlation was found between *oprD* expression and bacterial growth rate (rho=0.48, *P*=0.045), suggesting a link between porin expression and metabolic performance.

#### Intrastrain gene expression in the presence of IPM

Gene expression was determined in the selected isolates upon IPM exposure using an intrastrain comparison, by evaluating expression levels in the presence versus absence of the antibiotic (Fig. S10). In the presence of IPM, *oprD* was downregulated in 22% (4/18) of isolates and upregulated in 50% (9/18). Overexpression was observed for *mexA* in 39% (7/18), *mexY* in 67% (12/18), *mexC* in 61% (11/18) and *mexE* in 56% (10/18) of the isolates. The individual log_2_FoldChange values under IPM exposure and their interpretation are provided in Table S12.

No significant differences in gene expression were observed across susceptibility phenotypes (Fig. S11). However, *mexA* expression was significantly higher in PA14 isolates than in PAO1 isolates (*P*=0.0136). No significant differences were found for *oprD*, *mexC, mexE* or *mexY* across phylogroups (Fig. S11). Additionally, no correlation was found between gene expression and growth rate under IPM exposure.

#### Intrastrain gene expression in the presence of MEM

Gene expression in condition C (presence of MEM) was assessed for each of the selected isolates using an intrastrain comparison between expression levels in the presence and absence of MEM (Fig. S13).

The analysis revealed heterogeneity in the expression patterns of the evaluated genes (*oprD*, *mexA*, *mexY*, *mexC* and *mexE*), with both upregulation and downregulation observed across isolates. Under MEM exposure, *oprD* was downregulated in 28% of isolates and upregulated in 44% (8/18). *mexA* was overexpressed in 39% (7/18) of isolates, *mexY* in 28% (5/18), *mexC* in 56% (10/18) and *mexE* in 39% (7/18). The individual log_2_FoldChange values under MEM exposure and their interpretation are provided in Table S13.

No statistically significant associations were found between gene expression and susceptibility phenotype (*P*>0.05) (Fig. S13). Similarly, no significant differences in gene expression were observed across phylogroups under MEM exposure, in contrast to those detected under basal and IPM conditions (Fig. S14). No correlations were found between gene expression and growth rate in the presence of MEM (*P*>0.05).

#### Differential gene expression in the absence of antibiotics and in the presence of IPM or MEM

Differential gene expressions were evaluated under the described experimental conditions (A, B and C).

**Fig. 7.**
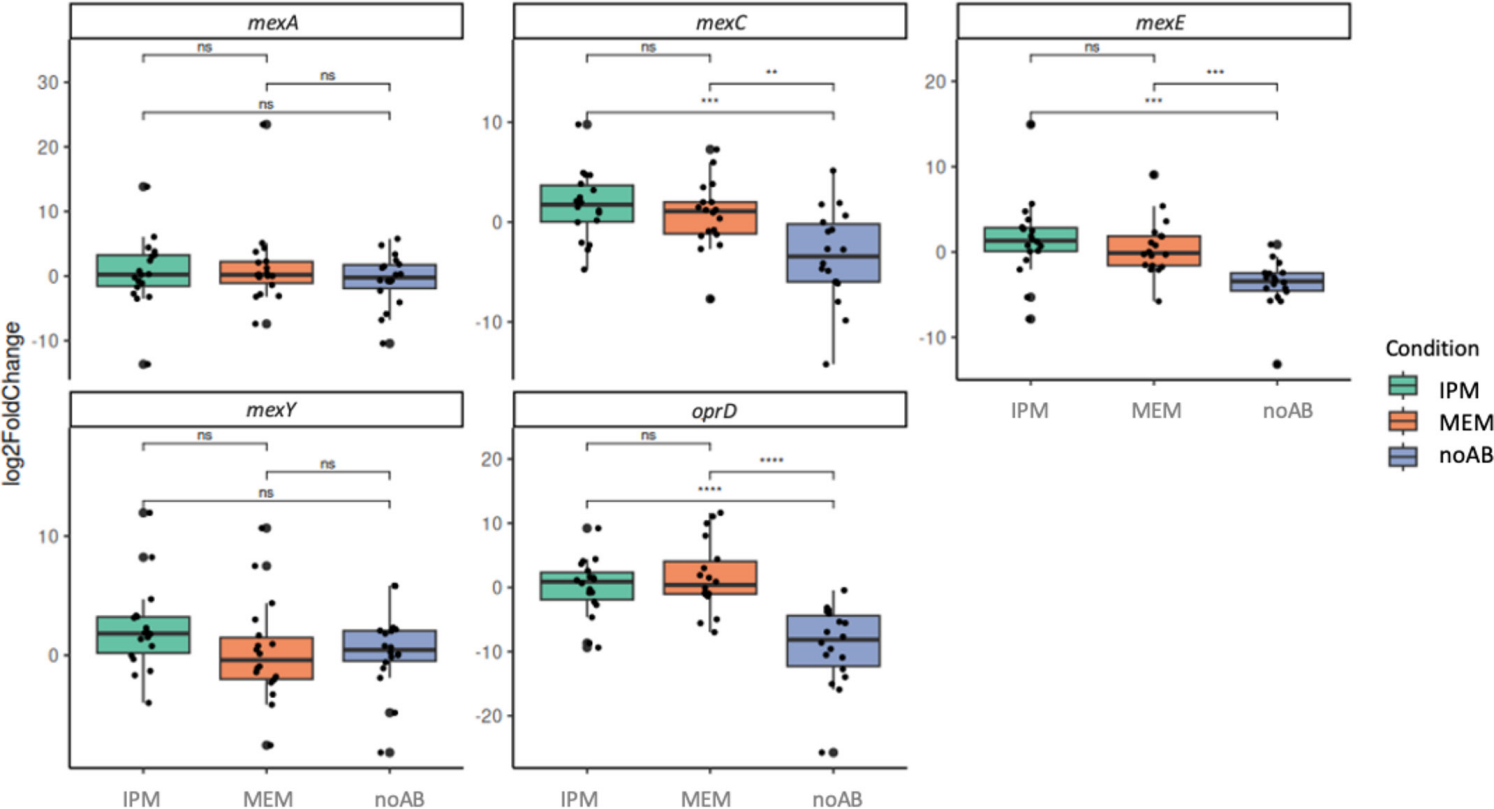
Boxplot of log_2_FoldChange in the absence of antibiotics and in the presence of imipenem and meropenem for each gene analysed. IPM, imipenem; MEM, meropenem; noAB, no antibiotic; ns, no significance.

For *mexA*, no statistically significant differences were observed across the tested conditions (*P*>0.05). In contrast, *mexC* showed significantly higher expression in the presence of IPM (*P*=0.026) and MEM (*P*=0.036) compared to the antibiotic-free condition. Similarly, *mexE* was significantly upregulated under MEM (*P*=0.0067) and IPM exposure (*P*=0.0039). No significant changes in expression were detected for *mexY* across the conditions (*P*>0.05). Regarding *oprD*, significant differences in expression were found between the antibiotic-free condition and both IPM (*P*=0.0012) and MEM (*P*=0.00061), suggesting transcriptional modulation of this gene in response to carbapenem exposure (Fig. 7).

#### Sequence variation in efflux pump regulatory genes

To further explore the genomic basis underlying the observed efflux pump expression patterns, sequence variation in the main regulators (*mexR*, *nalC*, *nalD*, *nfxB*, *mexS*, *mexT* and *mexZ*) of the analysed RND systems was examined (Table S14). Genotypic characterization of the regulatory genes identified synonymous, missense and frameshift variants; however, a strict genotype–phenotype correlation was not always evident (*P*>0.05). For instance, while frameshift variants in the *mexT* regulator (observed in isolates 70, 74, 92 and 318) generally coincided with lower or variable *mexE* expression, other isolates displayed elevated efflux pump transcription in the absence of obvious loss-of-function mutations in their respective repressors, suggesting the involvement of alternative regulatory pathways or strong induction by carbapenems.

## Discussion

Carbapenem resistance in *P. aeruginosa* arises from multiple genomic and adaptive mechanisms. The study of mechanisms of carbapenem resistance in *P. aeruginosa* has largely focused on the detection of CEGs, leaving a critical gap in the recognition of non-carbapenemase-mediated resistance mechanisms. This study aimed to characterize the genomic and phenotypic basis of carbapenem resistance in *P. aeruginosa* isolates collected from 28 healthcare centres across Mexico.

The phylogenomic analysis of 136 *P*. *aeruginosa* clinical isolates from healthcare centres in Mexico revealed a highly diverse population structure, clustering into the two major phylogroups previously described: PAO1 and PA14, while Ppa lineage was also identified. This phylogenomic clustering has been reported in genomic studies from other regions of the world, including Asia, Europe and Oceania [20,36], and reinforces the notion that *P. aeruginosa* maintains a conserved population structure regardless of geographic origin. Additionally, Ppa isolates displayed a greater genetic divergence as they formed a highly divergent clade, clearly separated from the rest of the clinical isolates, in addition to a lower ANI (<94%) and a higher abundance of sequence variants. This observation is in line with recent phylogenomic studies, suggesting that the former PA7 phylogroup does not belong to the core *P. aeruginosa* clade. A recent study [37] established the reclassification of PA7 as a distinct species based on genome-wide metrics, including ANI values below the accepted species threshold (<95%). Although historically considered an atypical *P. aeruginosa* strain, these results support the notion that PA7 falls outside the taxonomic boundaries of the *P. aeruginosa*. Its inclusion in this study, however, remains relevant for comparative purposes, given its frequent use as a reference genome in previous literature [20,36].

Among all STs identified, the most frequent was ST309 (29/136, 21%). This ST had previously been reported in Mexico in *P. aeruginosa* strains isolated from paediatric patients with bacteraemia between 2011 and 2014, where it was the most common (9/60, 15%) in the group of strains that showed high genetic variability [38]. ST309 has a widespread geographic distribution and has been reported in Asia, Europe and some countries in the Americas [39,42]. It is commonly associated with multidrug resistance and has been linked to the carriage of GES-type *β*-lactamases, some of which confer resistance to MEM [43]. The detection of ST309 across multiple hospitals in the present study further suggests potential interhospital dissemination or adaptation to the hospital environment, underscoring its epidemiological importance.

ST235 was identified in 14 of the 136 clinical isolates analysed. Although ST235 is a globally disseminated high-risk clone often associated with carbapenem resistance [19], its presence in Mexico has been rarely reported. The first documented identification of ST235 in Mexico was reported during an outbreak in a Mexican paediatric hospital, where 1 out of 15 clinical isolates collected in 2018 was identified as ST235 and classified as an extensively drug-resistant strain [19]. ST235 has been frequently reported in Colombia, where it is considered endemic [44]. Given the increasing transit of patients across borders, enhanced genomic surveillance of ST235 is warranted to monitor its potential spread and public health impact.

In addition, 13 isolates were assigned to ST111, another high-risk clone with global distribution [17,45]. A previous genomic surveillance study reported the presence of ST111 among five *P. aeruginosa* isolates collected from patients with urinary tract infections or pneumonia in Panama and Mexico, confirming its regional circulation [46]. This phylogenomic divergence was not only reflected in ST variation but also in the distribution of ARGs; for example, CEGs, including *bla*_GES_, *bla*_VIM_ and *bla*_IMP_, were found predominantly in PAO1 and PA14 lineages.

This study revealed clustering of isolates by phylogenetic lineage. Similar results were observed in a previous study conducted in Pakistan that analysed 142 *P*. *aeruginosa* isolates that showed differences in ARG profiles among phylogenetic clades, supporting the idea that the resistome is lineage-associated [18]. Together, these findings suggest that ARG content is shaped by the genomic background. Despite the detection of several CEGs, 62% of carbapenem-resistant isolates lacked any known CEGs. This proportion is consistent with a previous study that reported that up to 70% of CRPA isolates exhibit resistance through non-carbapenemase mechanisms [47]. Among these non-carbapenemase mechanisms, OprD disruption remains a key contributor. In this study, 75% of isolates harboured variants that potentially inactivate *oprD*, including nonsense variants, frameshifts and mobile element insertions, particularly in PAO1-related isolates. Such events are well-documented and represent a common path to carbapenem resistance through reduced outer membrane permeability [48].

The presence of nonsense and frameshift variants in *oprD* has been associated with an intermediate phenotype, particularly for IPM [47,49]. In the present study, isolates harbouring such variants displayed MICs in the intermediate range for IPM, while often remaining susceptible or exhibiting lower MICs for MEM. This result reflects the substrate specificity of OprD, which preferentially facilitates the uptake of IPM over MEM [50]. In contrast, isolates with OprD disruption and high-level resistance for both carbapenems (MIC>16 µg ml^−1^) also carried CEGs, suggesting that enzymatic degradation acts synergistically with porin inactivation to produce higher MICs. These findings support the fact that *oprD* disruption alone is insufficient to drive high resistance levels and likely represents an initial step in the resistance, later reinforced by horizontal acquisition of ARGs [51].

Growth kinetics were analysed to evaluate the physiological cost of carbapenem resistance in *P. aeruginosa*, particularly in growth efficiency under antibiotic pressure. The specific growth rate (h^−1^) did not differ significantly across all tested conditions (antibiotic-free and with IPM or MEM) and isolates with CEGs, suggesting that the presence of carbapenemase genes does not influence exponential growth once adaptation has occurred. In contrast, the addition of either IPM or MEM significantly prolonged the lag time compared to antibiotic-free conditions. This delay likely reflects stress response, or a metabolically adaptive phase triggered by carbapenem exposure [52].

Isolates that harboured CEGs maintained stable lag times across conditions, while those lacking CEGs exhibited a significant prolongation of lag time under carbapenem exposure. This result suggests that carbapenemase production confers a physiological advantage by facilitating immediate adaptation and enabling earlier transition to exponential growth. In contrast, isolates without CEGs may rely on inducible mechanisms such as efflux pump activation or porin regulation, resulting in a delayed response. These findings highlight that dynamic phenotypic metrics, such as lag time, can provide functional insights beyond static indicators like MICs or genotypic profiles. In this context, prolonged lag phases may reflect the metabolic burden of inducing resistance pathways in the absence of constitutive enzymatic protection [53,54].

In this study, under antibiotic-free conditions, *oprD* was consistently underexpressed across all isolates, while overexpression of the efflux pump genes *mexA* (38.89%), *mexC* (16.67%) and *mexY* (38.89%) was observed; in contrast, *mexE* overexpression was not detected. These findings differ from those reported in a study conducted in Egypt, where the expression of *oprD* and efflux pump genes was analysed in 634 clinical *P. aeruginosa* isolates. In that study, *oprD* underexpression was not observed, while overexpression of *mexA* (21.8%), *mexC* (75%), *mexE* (18.7%) and *mexY* (62%) was reported [31]. For instance, another study analysed the expression of *oprD* and *mexY* in 80 clinical isolates of CRPA under antibiotic-free conditions, and *oprD* underexpression (55%) and overexpression (2.5%) of *mexY* were detected [55]. The frequency of *oprD* underexpression and *mexY* overexpression was lower than that observed in this study, which could be due to genetic differences among the isolates analysed, possibly reflecting a generalized intrinsic regulation without the need for antibiotic exposure.

Additionally, the expression of the *oprD* and efflux pump genes (*mexA*, *mexC*, *mexE* and *mexY*) was analysed across the PAO1 and PA14 phylogroups, but also the Ppa lineage, and detected that the expression of *mexA* varied across phylogenetic lineages depending on the growth condition. Under antibiotic-free conditions, *mexA* expression was significantly higher in PAO1 isolates, suggesting a constitutive upregulation in this lineage. However, upon exposure to IPM, *mexA* expression became significantly higher in PA14 isolates. This shift implies that the regulatory networks controlling *mexA* expression may be differentially modulated by antibiotic stress depending on the phylogenetic background. While previous studies have reported regulatory differences between PAO1 and PA14 backgrounds [56], no studies were found that directly compared the inducible expression of *mexA* between phylogroups in clinical isolates under carbapenem exposure. However, a study conducted in a hospital in Mexico reported that nucleotide substitutions in *mexR*, the regulator gene of MexAB-OprM, were conserved within genetic lineages. In the present study, genetic lineages were also preserved, as missense variants in *mexR* were only found in isolates from the PAO1 phylogroup [57].

*oprD* expression correlated with growth rate and reflects the physiological role of OprD, which, in addition to being the main porin responsible for carbapenem uptake, also facilitates the transport of nutrients such as basic amino acids. Therefore, isolates with higher *oprD* expression may benefit from increased nutrient uptake, which could positively affect their growth rate. This result also suggests that loss or reduction of *oprD* expression, while contributing to resistance, may represent a biological cost in terms of metabolic efficiency or growth rate, as has been reported in other studies on the physiology of *P. aeruginosa* [12,51, 58].

Although OprD is recognized as the main entry porin for IPM in *P. aeruginosa*, and its loss or downregulation has been associated with carbapenem resistance, this study observed that some isolates showed *oprD* overexpression when exposed to IPM and MEM (22 and 28%, respectively), even when they displayed resistance to both antibiotics. This paradoxical overexpression could be due to a stress-induced response to antibiotic exposure.

Exposure to IPM may activate regulatory pathways that increase *oprD* transcription as part of an adaptive stress response. Similar patterns have been described in other contexts; antibiotic exposure induces the expression of permeability-related genes potentially as a compensatory mechanism for broader physiological alterations. However, increased *oprD* expression may not necessarily result in a functional protein. Some isolates analysed carry *oprD* variants that potentially alter their structure or function, which could explain the resistance phenotype observed despite transcriptional overexpression. Furthermore, the observed overexpression may represent a specific event within the bacterial stress response cycle and not reflect a stable adaptation over time. This finding underscores the need for longitudinal studies including multiple time points to characterize *oprD* regulatory patterns more precisely in response to antibiotic pressure [12,58, 59].

An increase in the number of isolates overexpressing efflux pump genes in the presence of either of the two carbapenems was observed. It has been reported that the expression of efflux pump genes can be modulated by environmental stress factors, such as nutritional limitation or antimicrobial exposure [60], and this fact may explain the overexpression. However, to date, no studies have evaluated the expression of efflux pump genes in the presence of carbapenems in clinical isolates of *P. aeruginosa*.

In addition to the carbapenem mechanisms described above, the contribution of chromosomal AmpC deregulation should be considered. Although carbapenems are relatively stable against AmpC-mediated hydrolysis, derepression of the chromosomal AmpC *β*-lactamase has been shown to influence *β*-lactam susceptibility profiles [61]. While AmpC expression was not evaluated in this study, derepression of AmpC may contribute to reduced carbapenem susceptibility when combined with porin loss, reinforcing the multifactorial nature of carbapenem resistance in this species.

This study has some limitations. First, antibiotic concentrations were tailored to the individual MICs of each strain, which may limit direct comparisons of the stimulatory effects of imipenem and meropenem on *oprD* and efflux pump gene expression across isolates. Second, the sample size for gene expression analysis was relatively small (*n*=18), which reduces the statistical power of the analyses and limits the ability to detect subtle correlations. Larger cohorts would be necessary to strengthen the robustness and generalizability of these observations. Finally, although clinical isolates were studied, findings were not correlated with clinical data.

In conclusion, clinical *P. aeruginosa* isolates clustered into PAO1 and PA14 phylogroups with ST309 being the most frequent ST. In addition, isolates belonging to the Ppa lineage were identified within this carbapenem-resistant isolates group. Resistance gene content showed phylogenetic clustering, suggesting a lineage-dependent acquisition of resistance determinants. Potentially inactivating *oprD* variants were associated with carbapenem resistance phenotypes, while missense variants were more frequent in susceptible isolates. Isolates with CEGs had stable lag phases under antibiotic exposure, whereas CEG-negative isolates displayed prolonged lag phases, indicating a physiological cost. The expression of *mexA* was significantly higher in PAO1 isolates under antibiotic-free condition but increased notably in PA14 isolates upon IPM exposure, while upregulation of *mexA*, *mexC* and *mexY* was induced by exposure to carbapenems. These findings highlight the complex, lineage-induced interplay between carbapenemases and adaptive resistance mechanisms in *P. aeruginosa*.

## Supplementary material

10.1099/mgen.0.001639 Uncited Supplementary Material 1.

## Abbreviations

ANI: average nucleotide identity
ARGs: antimicrobial resistance genes
CEG: carbapenemase-encoding gene
CRPA: carbapenem-resistant *Pseudomonas aeruginosa*
MIC: minimum inhibitory concentration
MLST: multilocus sequence typing
RND: resistance–nodulation–division
ST: sequence type
