# Supplementary material for: Phylogenomic and phenotypic profiling of carbapenem-resistant Pseudomonas aeruginosa clinical isolates reveals lineage-specific resistance mechanisms and adaptive responses
Source: Microb Genom. 2026 Feb 9;12(2):001639. doi: 10.1099/mgen.0.001639 (PMC12888230; doi:10.1099/mgen.0.001639)
Supplement: Uncited Supplementary Material 1. [file mgen-12-01639-s001.pdf]

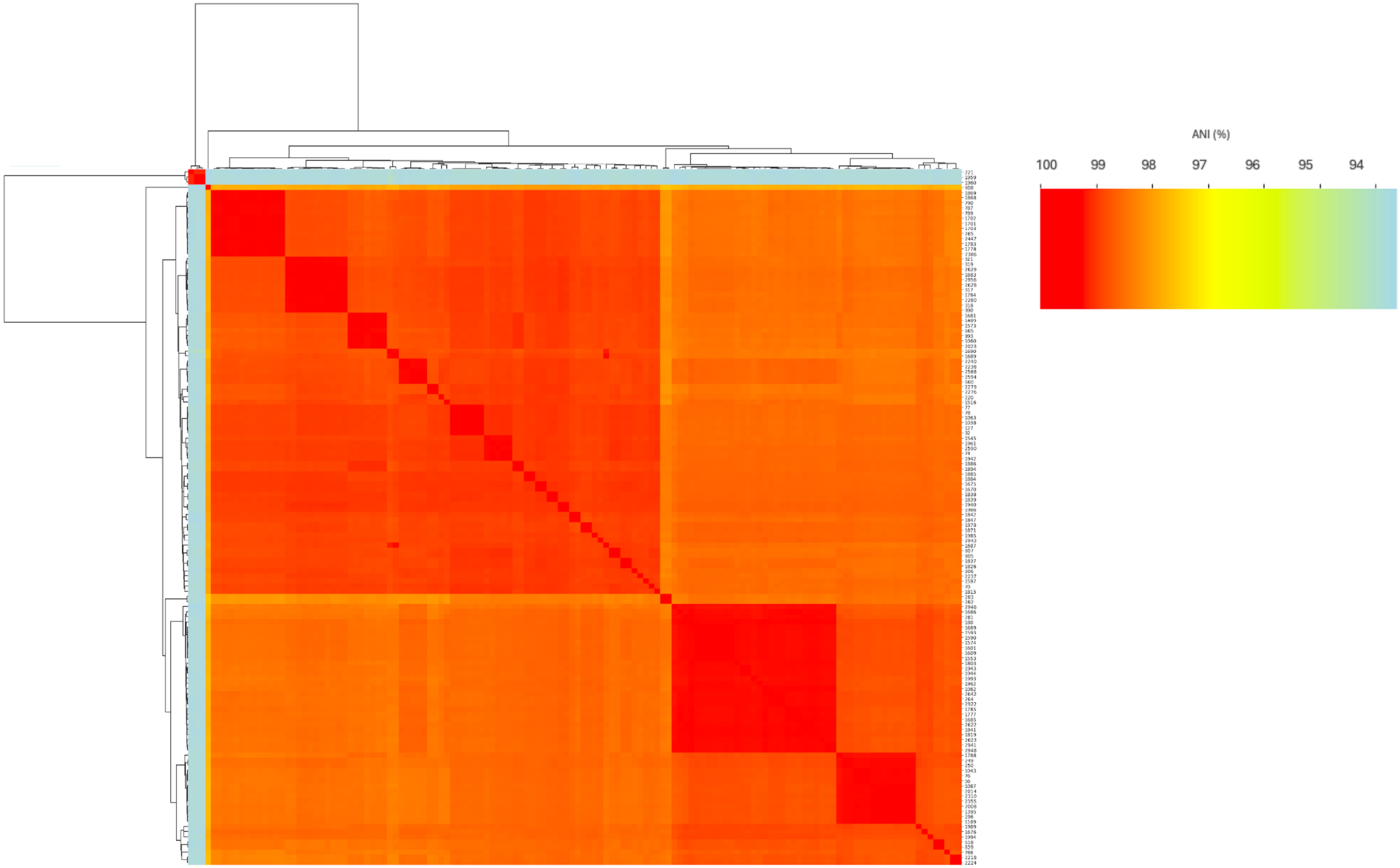

Figure S1. Heatmap representing pairwise average nucleotide identity (ANI) values calculated from whole-genome sequences of the 136 clinical *P. aeruginosa* isolates.

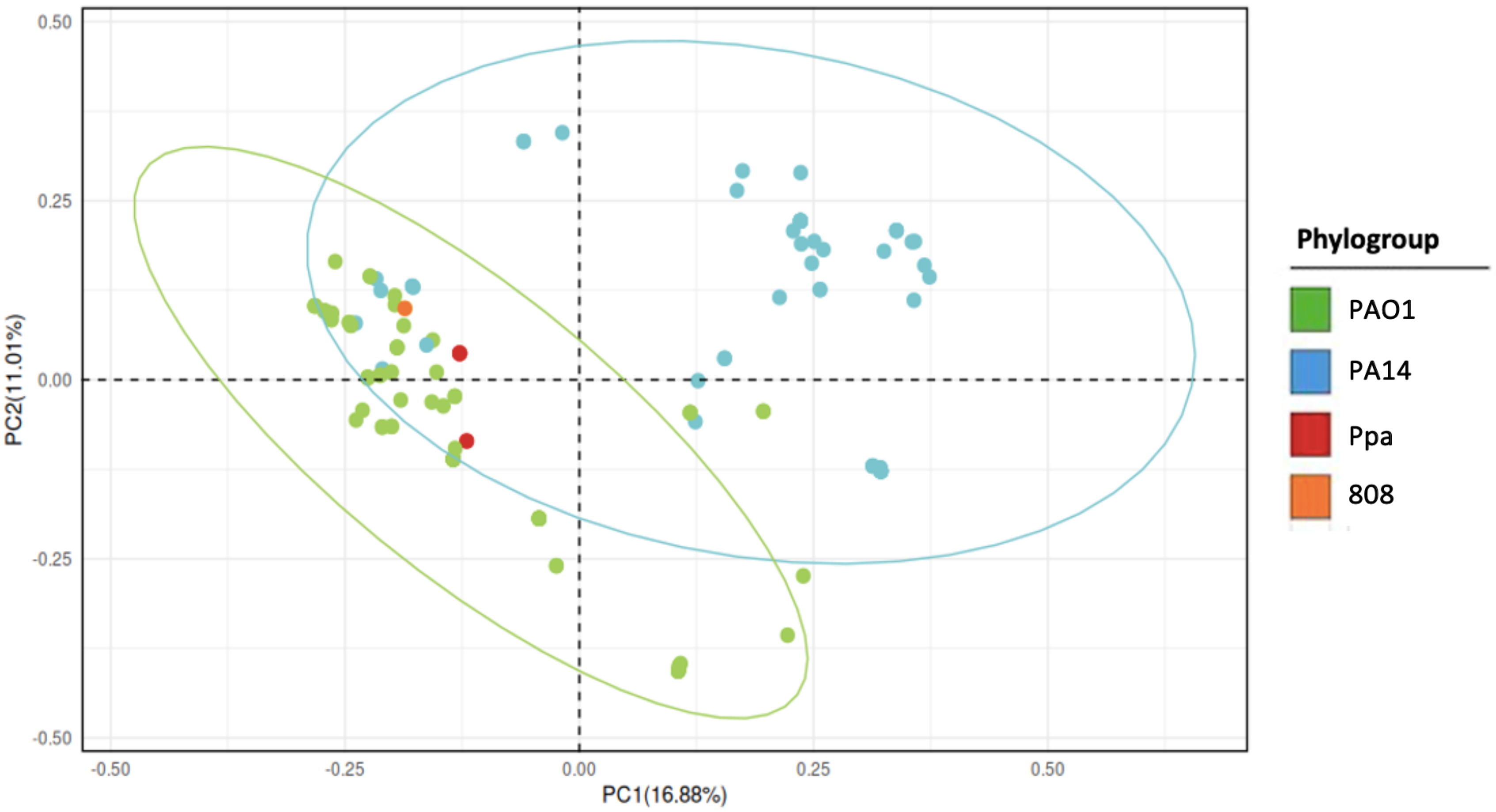

Figure S2. Principal Coordinates Analysis (PCoA) of acquired resistance genes in *P. aeruginosa* isolates. The analysis is based on a binary presence/absence matrix of resistance genes, with points colored according to phylogenomic group (PAO1, PA14, and Ppa). Principal components PC1 and PC2 explain 16.88% and 11.01% of the variance, respectively. Ppa: *P. paraeruginosa*.

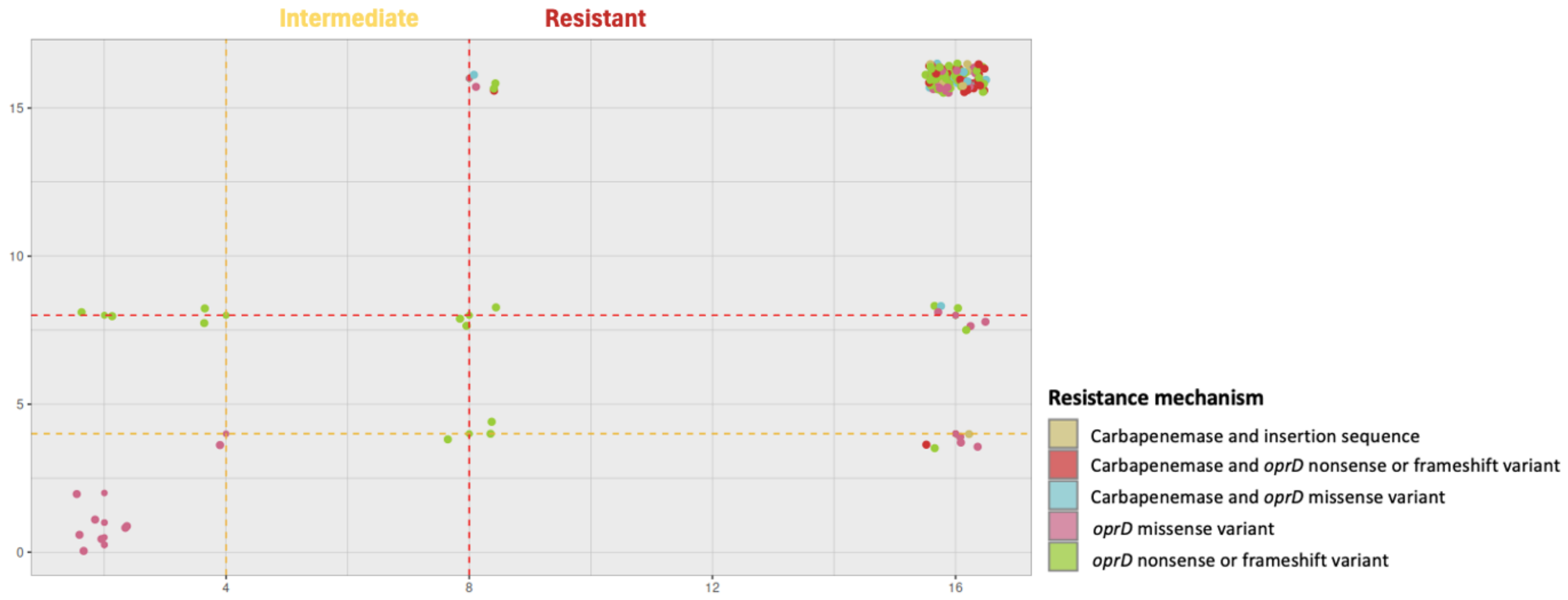

Figure S3. Correlation between genetic resistance mechanisms and minimum inhibitory concentration (MIC) values for imipenem and meropenem in the analyzed *P. aeruginosa* isolates.

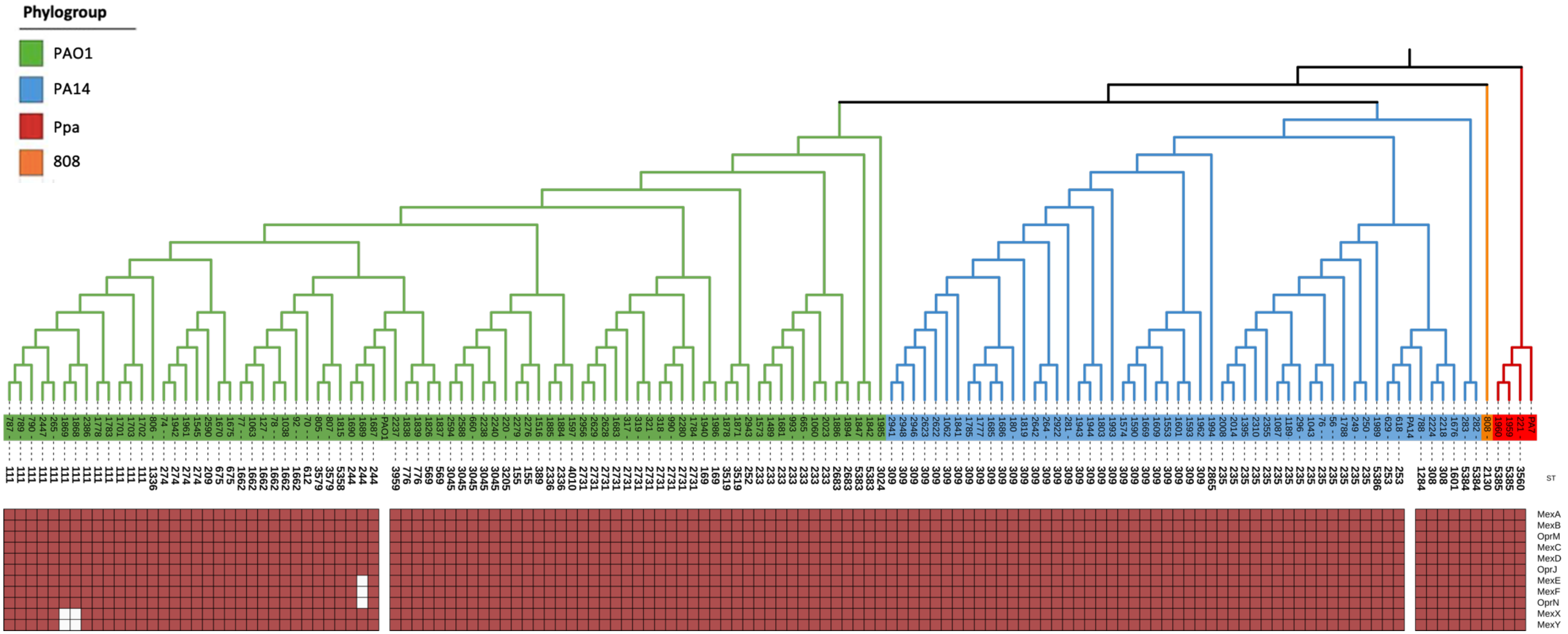

Figure S4. Presence of resistance-nodulation-cell division (RND) family efflux pumps in the analyzed *P. aeruginosa* strains. Phylogroups are represented by color green (PAO1), blue (PA14) and red (Ppa). Ppa: *P. paraeruginosa*.

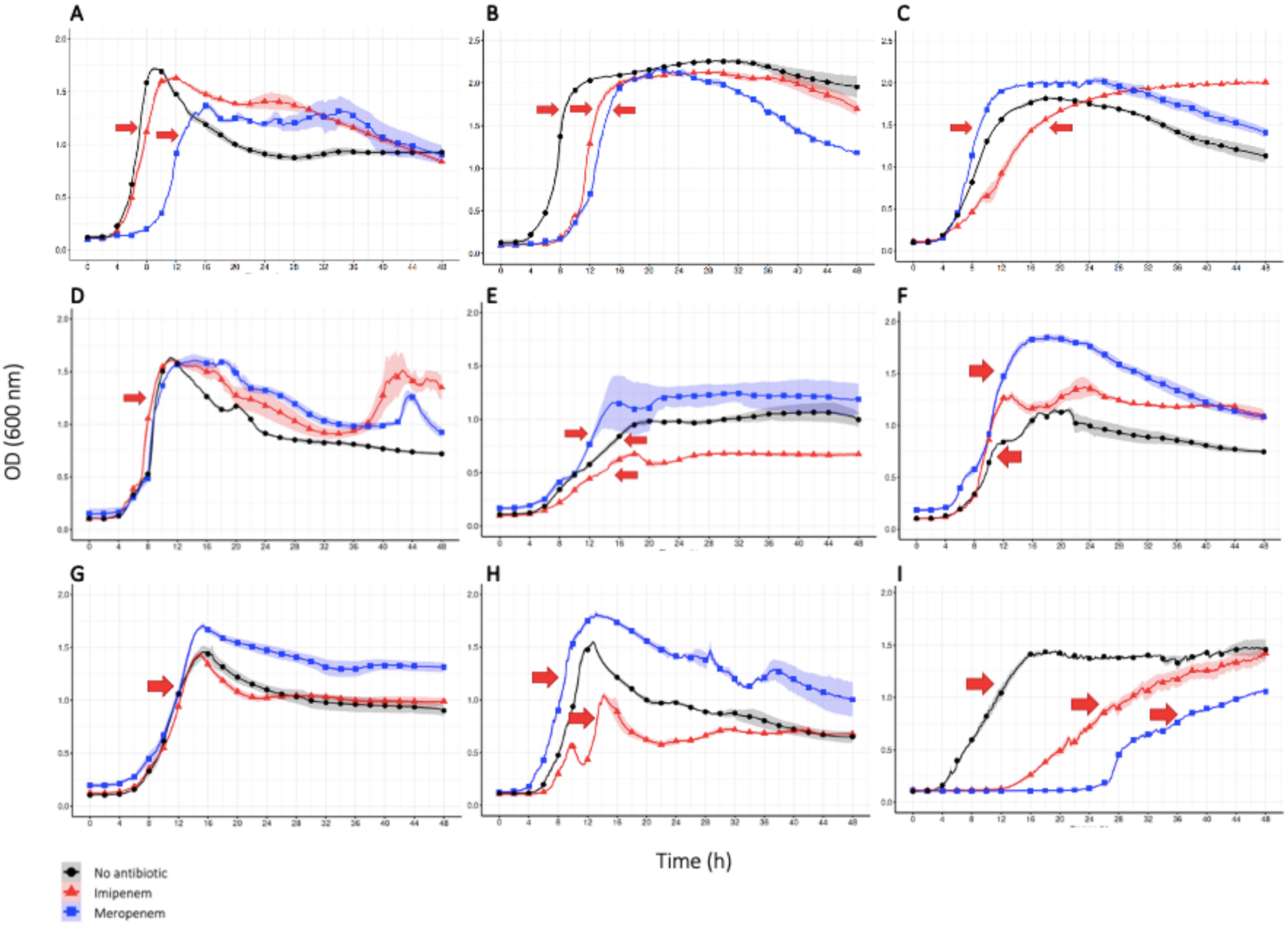
A
B
C
D
E
F
G
H
I
OD (600 nm)
Time (h)
No antibiotic
Imipenem
Meropenem

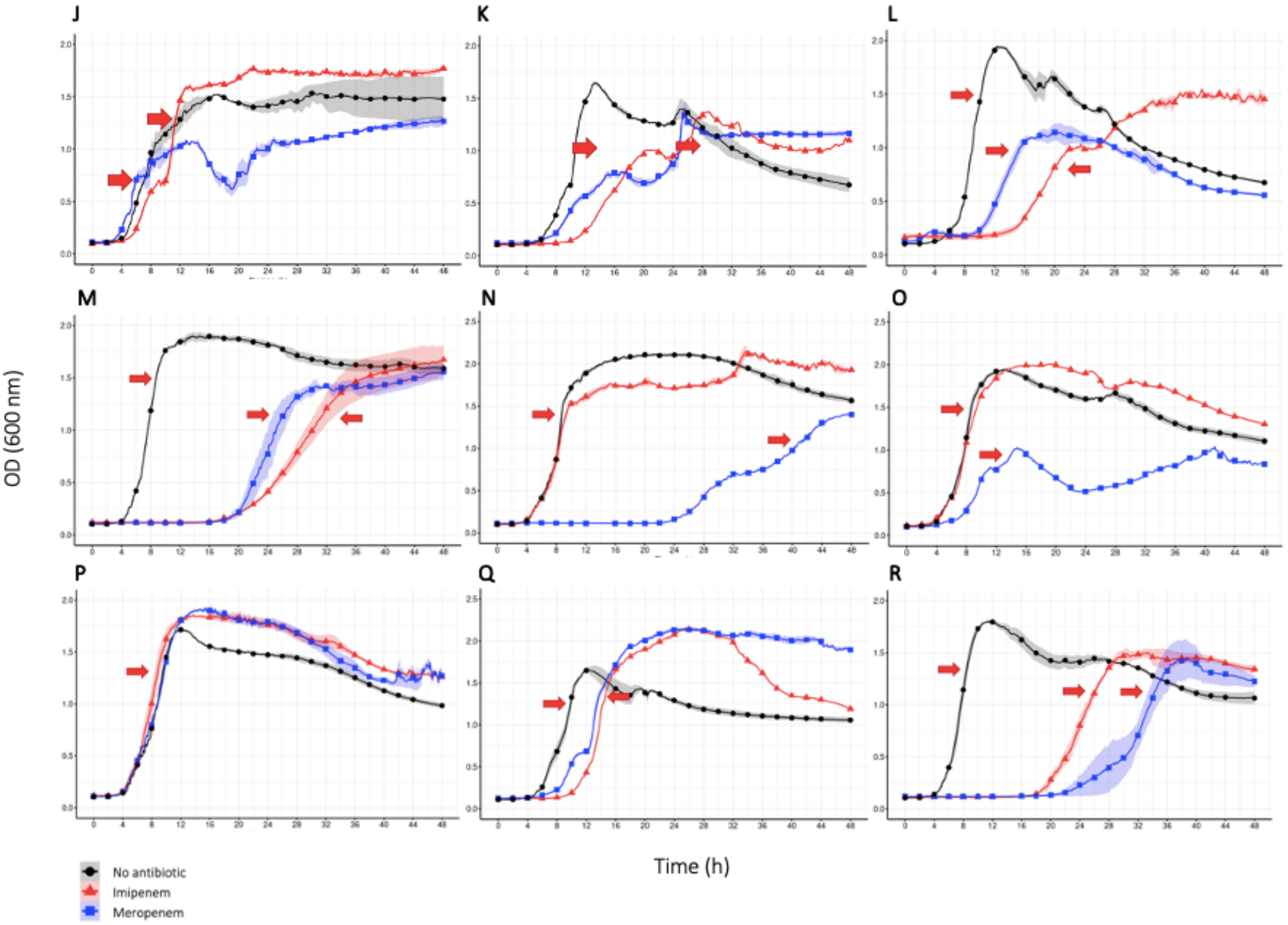

J
K
L
M
N
O
P
Q
R
OD (600 nm)
Time (h)
No antibiotic
Imipenem
Meropenem

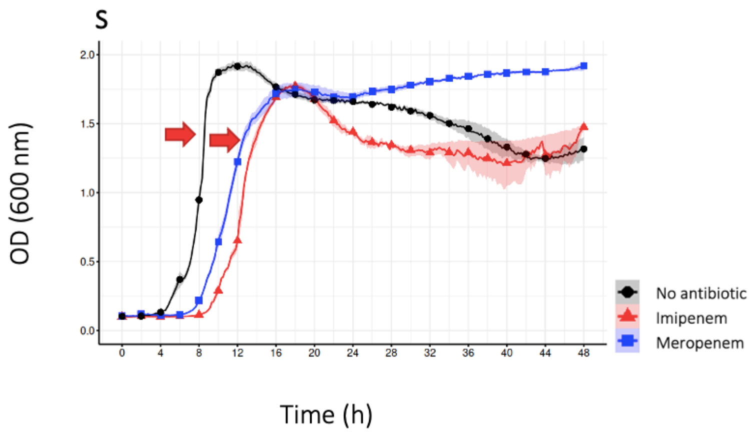

Figure S5. Growth curves of the *P. aeruginosa* isolates included in the gene expression analysis. Each panel (A–S) shows OD_{600} over 48 hours under three conditions: without antibiotic (black), with imipenem (red), and with meropenem (blue). Curves represent biological triplicates. Isolates marked with an asterisk (*) harbor carbapenemase-encoding genes. Isolates by panel: A: 70; B: 74; C: 221; D: 281; E: 318 *; F: 787*; G: 990 *; H: 1778*; I: 1841; J: 1871*; K: 1878; L: 1940; M: 1942; N: 1962; O: PAO1; P: 92 *; Q: 2948*; R: 2218; S: 1959.

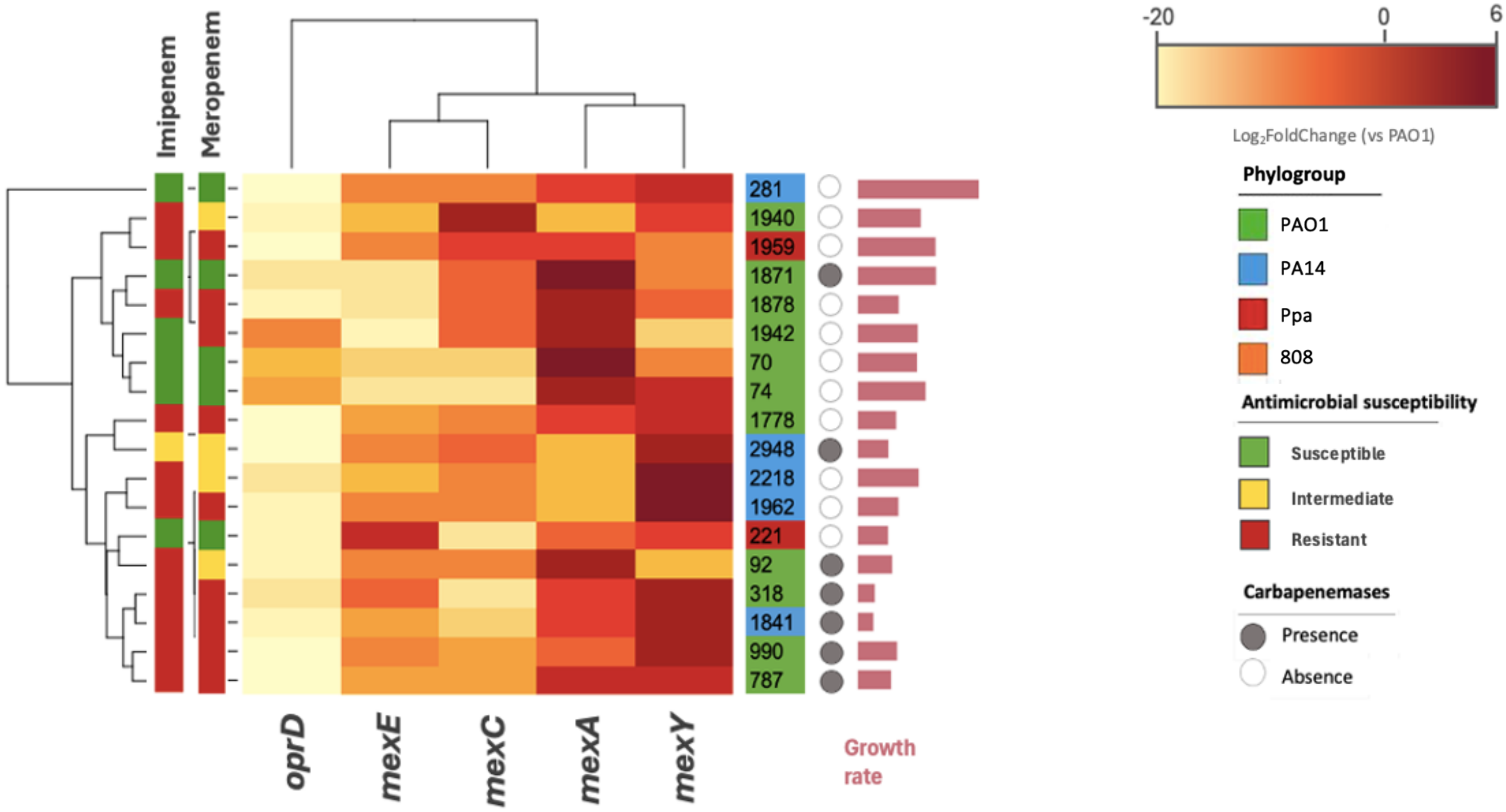

Figure S6. Heatmap showing the log₂FoldChange in relative gene expression for each strain exposed to imipenem, compared to the same strain under no antibiotic condition. Ppa: *P. paraeruginosa*.

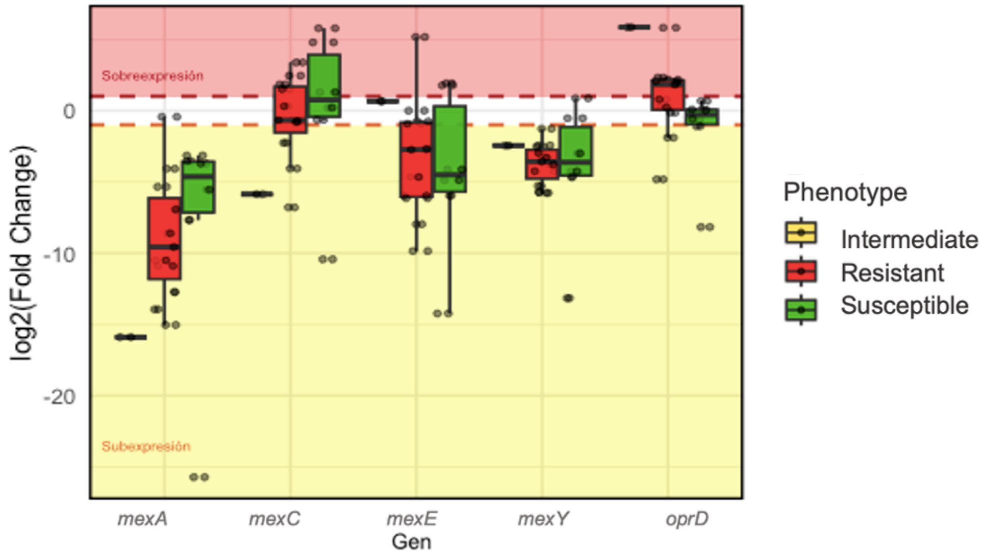

Figure S7. Boxplot of log_2FoldChange in gene expression according to susceptibility profile (susceptible, intermediate, resistant) for each gene analyzed in no antibiotic condition. No statistically significant differences were observed ($p > 0.05$).

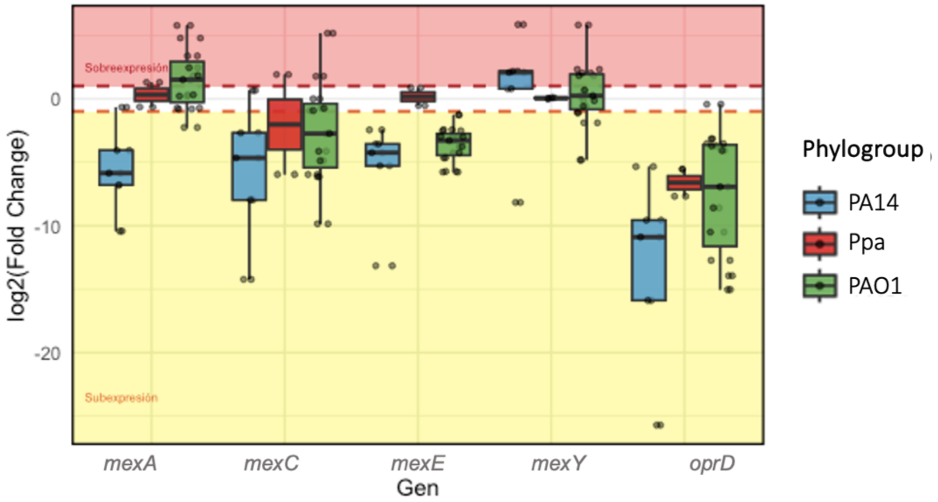

Figure S8. Boxplot of log_2FoldChange in gene expression across phylogroups (PAO1, PA14, and Ppa) for each gene analyzed in no antibiotic condition. A statistically significant difference was found in the expression of *mexA* between phylogroups PAO1 and PA14 ($p = 0.0086$). Ppa: *P. paraeruginosa*.

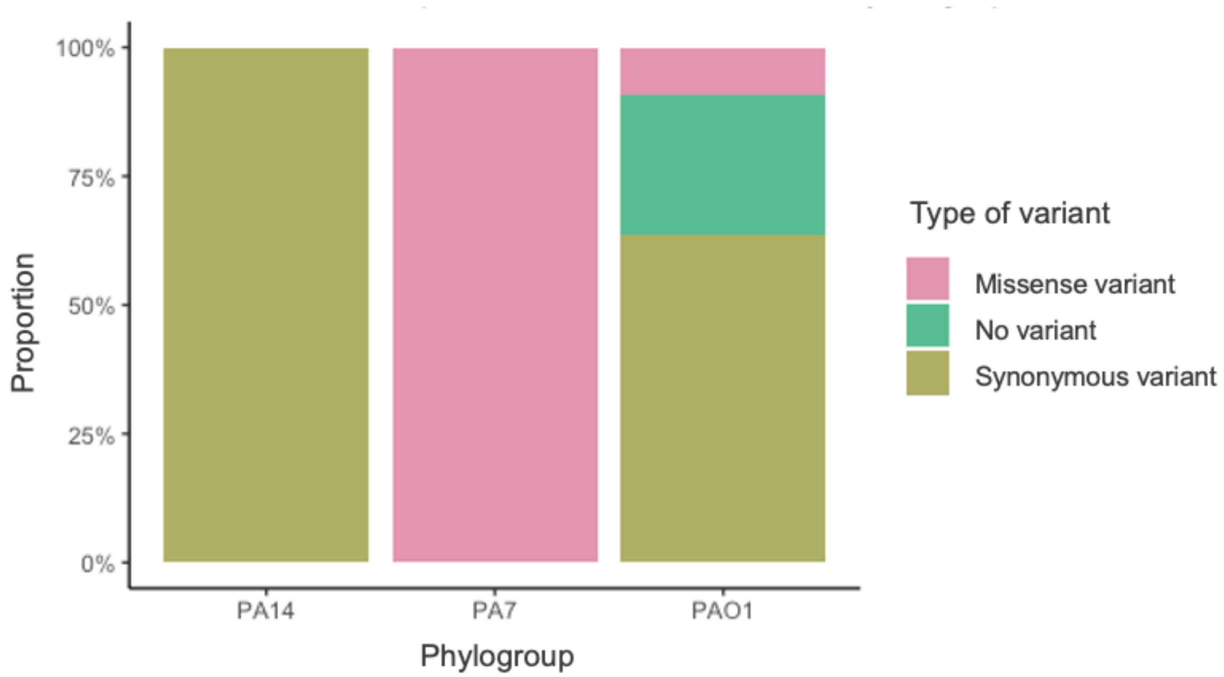

Figure S9. Distribution of mutation types in the mexR repressor gene across phylogroups. The mutation pattern varied significantly between phylogroups (p = 0.0086). Ppa: *P. paraeruginosa*.

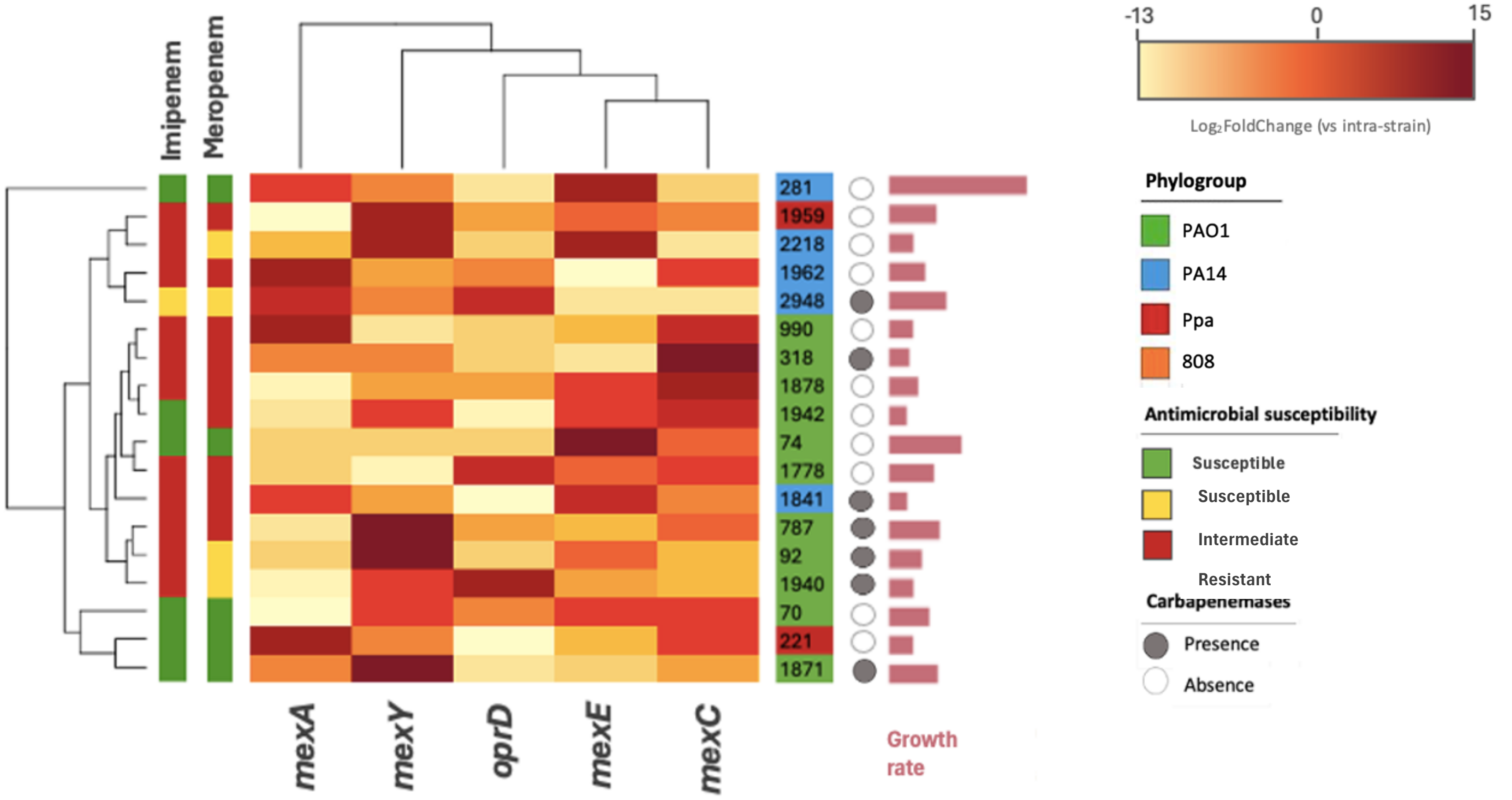

Figure S10. Heatmap showing the relative gene expression (log₂FoldChange) of each strain exposed to imipenem, compared to the same strain in the absence of the antibiotic. Ppa: *P. paraeruginosa*.

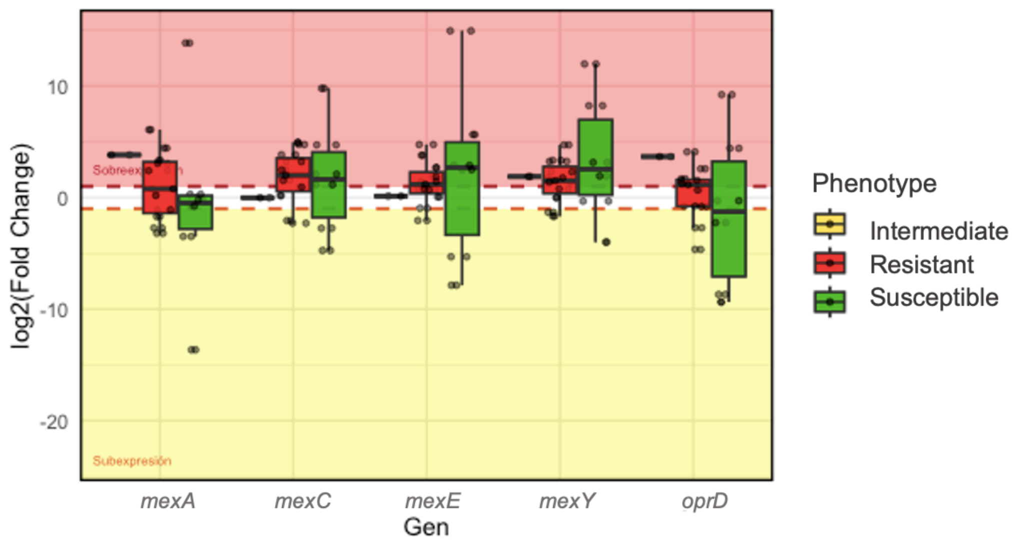

Figure S11. Boxplot of log_2FoldChange in gene expression by susceptibility profile (susceptible, intermediate, resistant) for each gene analyzed under imipenem exposure. No statistically significant differences were found ($p > 0.05$).

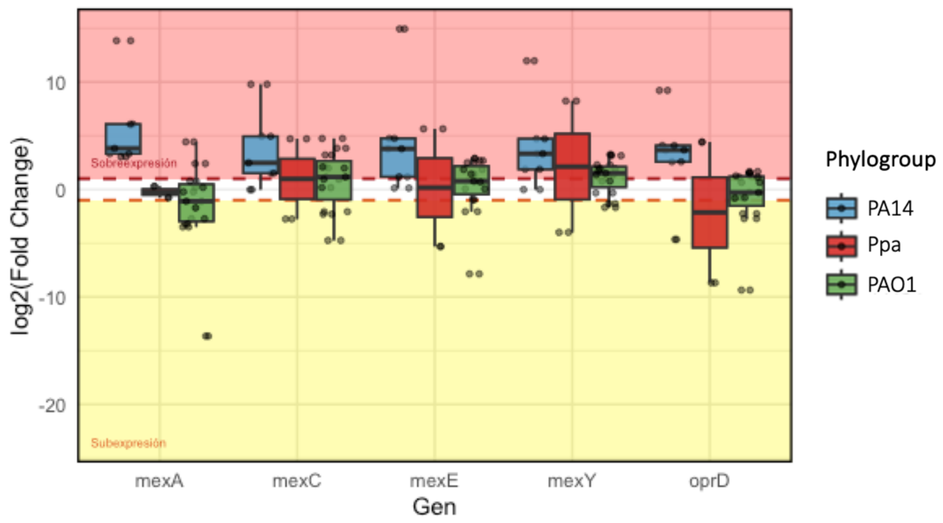

Figure S12. Boxplot of log_2FoldChange in gene expression by phylogroup (PAO1, PA14, Ppa) under imipenem exposure. A statistically significant difference was found in *mexA* expression between phylogroups PAO1 and PA14 ($p = 0.0136$). Ppa: *P. paraeruginosa*.

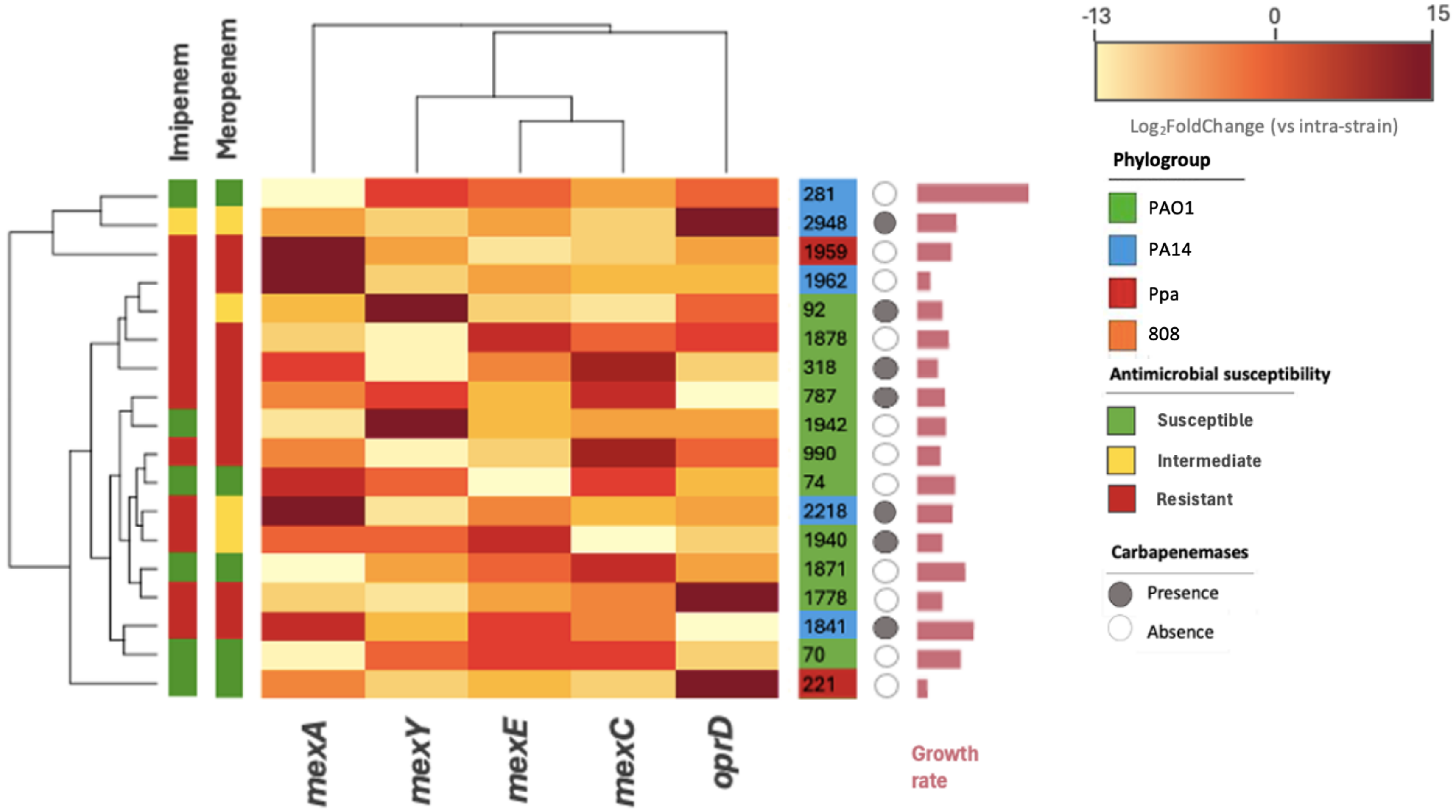

Figure S13. Heatmap showing the $\log_2$ fold change in relative gene expression for each strain under exposure to meropenem, compared to the same strain in the absence of the antibiotic. Ppa: *P. paraeruginosa*.

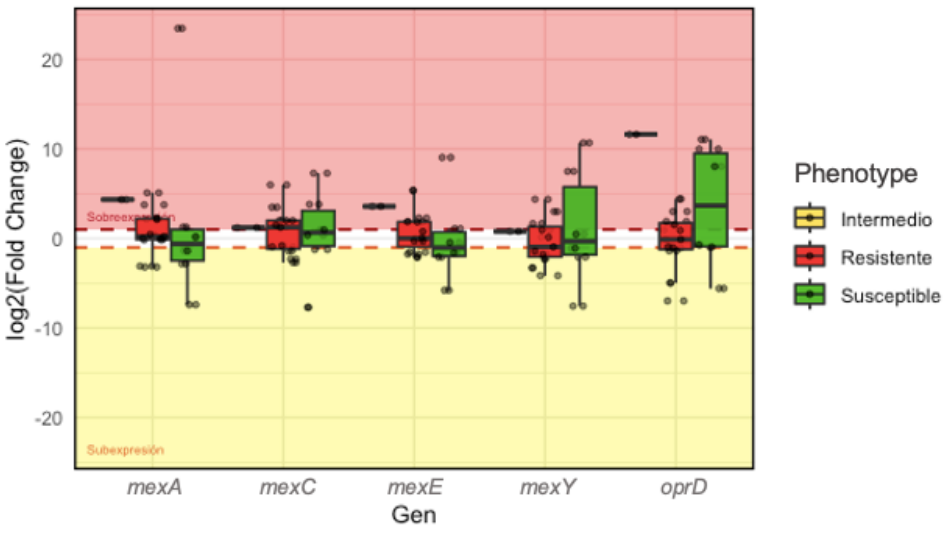

Figure S14. Boxplot of log_2(Fold Change) in gene expression by susceptibility profile under meropenem exposure for each gene analyzed. No statistically significant differences were observed between phenotypes and gene expression levels ($p > 0.05$).

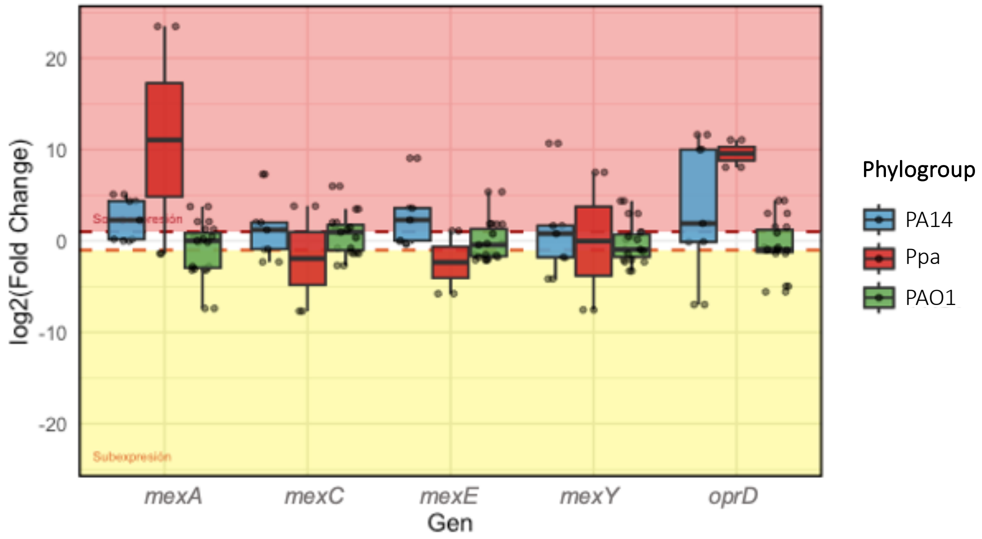

Figure S15. Boxplot of log_2(Fold Change) in gene expression by phylogroup (PAO1, PA14, Ppa) under meropenem exposure. No statistically significant differences were observed between phylogroups and gene expression levels ($p > 0.05$). Ppa: *P. paraeruginosa*.

Table S1. Access numbers for each genome sequenced in this study.

Access number	Sample ID	Access number	Sample ID	Access number	Sample ID	Access number	Sample ID
SRX27374837	1885	SRX27374803	1681	SRX27374769	1942	SRX27374735	2629
SRX27374836	1884	SRX27374802	92	SRX27374768	1940	SRX27374734	2628
SRX27374835	220	SRX27374801	1676	SRX27374767	1894	SRX27374733	2623
SRX27374834	1878	SRX27374800	1675	SRX27374766	1886	SRX27374732	2622
SRX27374833	1871	SRX27374799	1670	SRX27374765	807	SRX27374731	2594
SRX27374832	1869	SRX27374798	1669	SRX27374764	806	SRX27374730	2590
SRX27374831	1868	SRX27374797	1609	SRX27374763	805	SRX27374729	2588
SRX27374830	1847	SRX27374796	1601	SRX27374762	790	SRX27374728	2447
SRX27374829	1842	SRX27374795	1597	SRX27374761	76	SRX27374727	2386
SRX27374828	1841	SRX27374794	1593	SRX27374760	789	SRX27374726	250
SRX27374827	1839	SRX27374793	1590	SRX27374759	788	SRX27374725	2355
SRX27374826	1838	SRX27374792	1574	SRX27374758	787	SRX27374724	2310
SRX27374825	1837	SRX27374791	78	SRX27374757	665	SRX27374723	2280
SRX27374824	180	SRX27374790	1573	SRX27374756	660	SRX27374722	2279
SRX27374823	1826	SRX27374789	1553	SRX27374755	629	SRX27374721	2276
SRX27374822	1819	SRX27374788	1545	SRX27374754	618	SRX27374720	2240
SRX27374821	1815	SRX27374787	1516	SRX27374753	321	SRX27374719	2238
SRX27374820	1803	SRX27374786	1489	SRX27374752	319	SRX27374718	2237
SRX27374819	1788	SRX27374785	1395	SRX27374751	318	SRX27374717	2224
SRX27374818	1785	SRX27374784	1189	SRX27374750	74	SRX27374716	2218

SRX27374817	1784	SRX27374783	1087	SRX27374749	317	SRX27374715	249
SRX27374816	1783	SRX27374782	1063	SRX27374748	296	SRX27374714	2023
SRX27374815	1778	SRX27374781	1062	SRX27374747	283	SRX27374713	2014
SRX27374814	1777	SRX27374780	77	SRX27374746	282	SRX27374712	2008
SRX27374813	127	SRX27374779	1060	SRX27374745	281	SRX27374711	1994
SRX27374812	1703	SRX27374778	1043	SRX27374744	265	SRX27374710	1993
SRX27374811	1702	SRX27374777	1038	SRX27374743	2956	SRX27374709	1989
SRX27374810	1701	SRX27374776	993	SRX27374742	2948	SRX27374708	1986
SRX27374809	1690	SRX27374775	990	SRX27374741	2946	SRX27374707	1985
SRX27374808	1689	SRX27374774	808	SRX27374740	2943	SRX27374706	1962
SRX27374807	1687	SRX27374773	1960	SRX27374739	2941	SRX27374705	1961
SRX27374806	1686	SRX27374772	1959	SRX27374738	2922	SRX27374704	221
SRX27374805	1685	SRX27374771	1944	SRX27374737	264	SRX27374703	70
SRX27374804	1683	SRX27374770	1943	SRX27374736	2642	SRX27374702	56

Table S2. Oligonucleotide sequences used in this study.

**Gen**	**Sequence 5' → 3'**
oprD	F:CTACGGCTACGGCGAGGAT R:GACCGGACTGGACCACGTACT
mexA	F:AACCCGAACAACGAGCTG R:ATGGCCTTCTGCTTGACG
mexC	F:GGAAGAGCGACAGGAGGC R:CTGCACCGTCAGGCCCTC
mexE	F:TACTGGTCCTGAGCGCCT<br>R:CAGCGGTTGTTCGATGA
mexY	F:CCGCTACAACGGCTATCCCT R:AGCGGGATCGACCAGCTTTC
rpsL	F:CCGCTACAACGGCTATCCCT R:AGCGGGATCGACCAGCTTTC

Hassuna NA, Darwish MK, Sayed M, et al. Molecular epidemiology and mechanisms of high-level resistance to meropenem and imipenem in *Pseudomonas aeruginosa*. Infect Drug Resist. 2020:285-93.

Table S3. Summary of statistical analyses performed.

**Comparison / Analysis**	**Test Used**	**Post-hoc / Correction**
Normality of growth rate (h^{-1}), lag time (h), and gene expression data (log_2FoldChange of *oprD*, *mexA*, *mexC*, *mexE*, *mexY*)	Shapiro–Wilk test	–
Growth rate (h^{-1}), lag time of isolates with and without carbapenemase-encoding genes (h) and expression of each gene (log_2FoldChange of *oprD*, *mexA*, *mexC*, *mexE*, *mexY*) across conditions (no antibiotic, imipenem, meropenem)	ANOVA for repeated measures (if parametric) / Friedman test (if non-parametric)	t-test or Wilcoxon signed-rank test with Bonferroni correction
Lag time difference between isolates with and without carbapenemase-encoding genes within each condition (no antibiotic, imipenem, and meropenem)	Unpaired t-test or Mann–Whitney	-
Association between gene expression and carbapenem susceptibility phenotype (S/I/R)	Kruskal–Wallis test	–
Comparison of gene expression across phylogroups (PAO1, PA14, PA7) in each condition	Kruskal–Wallis test	Dunn's test with Bonferroni correction
Association between *mexR* mutations and *mexA* overexpression across phylogroups	Chi-square test	–
Correlation between growth rate and gene expression (*oprD*, *mexA*, *mexC*, *mexE*, *mexY*)	Spearman correlation	–
Correlation between resistance genes and phylogoup	Principal Coordinates Analysis (PCoA)	-

For all tests, a $p < 0.05$ was considered statistically significant.

Table S4. Minimum inhibitory concentration (µg/mL) for each antibiotic for the study strains.

Clinical isolate	TZP	CAZ	FEP	CZA	CT	IPM	MEM	AMK	TOB	CIP	LVX	ATM	CL
56	>128	>64	>32	>16	>32	>16	>16	>64	6	>4	6	6	<2
70	8	2	2	2	0.5	2	<0.25	2	22	0.5	23	26	<2
74	16	8	8	2	1	2	0.5	4	20	0.25	23	22	<2
76	>128	>64	>32	>16	>32	>16	>16	>64	6	>4	6	6	<2
77	>128	>64	>32	>16	>32	>16	>16	>64	14	>4	6	25	<2
78	>128	>64	>32	>16	>32	>16	>16	>64	13	>4	6	24	<2
92	64	>64	>32	>16	>32	>16	4	>64	14	>4	6	24	<2
127	64	>64	>32	>16	>32	>16	16	>64	14	>4	6	23	<2
180	>128	>64	>32	>16	>32	>16	>16	>64	6	>4	6	6	<2
220	32	2	8	2	0.5	>16	>16	2	24	0.5	19	17	<2
221	<4	2	2	2	1	2	<0.25	2	21	0.25	26	24	<2
249	64	32	8	8	1	>16	>16	8	6	>4	6	20	<2
250	64	32	8	8	1	8	>16	8	6	>4	6	18	<2
264	8	16	8	2	1	>16	>16	>64	6	>4	6	25	<2
265	>128	>64	16	>16	>32	>16	>16	>64	6	>4	6	21	<2
281	64	32	>32	2	>32	2	<0.25	>64	6	1	15	24	<2
282	8	2	4	1	0.5	2	2	1	24	>4	12	27	<2
283	16	4	8	2	1	>16	8	1	27	2	10	24	<2
296	>128	>64	>32	>16	>32	>16	>16	>64	6	>4	6	6	<2
317	>128	>64	>32	>16	>32	>16	>16	>64	6	>4	6	12	<2
318	64	4	16	2	1	>16	>16	>64	6	>4	6	6	<2
319	>128	>64	>32	>16	>32	>16	>16	>64	6	>4	6	10	<2
321	>128	>64	>32	>16	>32	>16	>16	>64	6	>4	6	10	<2
618	8	2	2	2	1	8	8	2	23	0.12	26	27	<2
629	8	2	2	2	1	8	4	4	22	0.25	27	25	<2
660	64	>64	>32	8	>32	>16	>16	>64	6	>4	6	15	<2
662	64	>64	>32	8	>32	>16	>16	>64	6	>4	6	12	<2
665	>128	>64	>32	>16	>32	>16	>16	>64	6	>4	6	25	<2
787	>128	32	16	>16	>32	>16	>16	>64	6	>4	6	23	<2
788	>128	>64	>32	>16	>32	>16	8	>64	6	>4	6	6	<2
789	32	8	16	>16	>32	>16	>16	>64	6	>4	6	23	<2
790	32	16	16	>16	>32	>16	>16	16	6	>4	6	26	<2
805	8	2	2	2	0.5	8	8	2	30	0.062	42	32	<2
806	>128	32	16	4	4	>16	>16	2	16	0.25	27	31	<2
807	16	2	2	2	0.5	8	>16	1	28	0.5	20	22	<2
808	32	2	8	4	1	>16	>16	4	28	1	16	21	<2
990	>128	>64	>32	>16	>32	>16	>16	>64	6	>4	6	6	<2
993	>128	32	>32	>16	>32	>16	>16	>64	6	>4	6	33	<2
1038	64	>64	>32	>16	>32	>16	>16	>64	22	>4	6	31	<2
1043	>128	>64	>32	>16	>32	>16	>16	>64	6	>4	14	6	<2
1060	>128	32	>32	>16	>32	>16	>16	>64	6	>4	6	31	<2
1062	>128	32	>32	>16	>32	>16	>16	>64	6	>4	14	6	<2
1063	64	>64	>32	>16	>32	>16	>16	>64	22	>4	6	31	<2
1087	>128	32	>32	>16	>32	>16	>16	>64	6	>4	6	6	<2
1189	>128	32	>32	>16	>32	>16	>16	>64	6	>4	10	6	<2
1395	>128	32	>32	>16	>32	>16	>16	>64	6	>4	6	31	<2
1489	>128	32	16	>16	>32	>16	>16	>64	6	>4	12	6	<2
1516	32	16	2	2	4	>16	>16	>64	6	0.5	34	36	<2
1545	16	2	8	2	1	2	1	4	28	0.125	38	28	<2
1553	8	2	2	2	1	>16	>16	>64	10	0.125	36	27	<2
1573	>128	32	>32	>16	>32	>16	>16	>64	6	>4	6	30	<2
1574	8	2	2	2	1	>16	>16	32	10	0.125	43	36	<2
1590	8	2	2	2	1	>16	>16	>64	6	0.125	42	43	<2
1593	8	2	2	2	1	>16	>16	>64	6	0.125	44	41	<2
1597	32	2	4	4	0.5	4	8	1	33	0.5	26	28	<2
1601	8	2	2	2	1	>16	>16	>64	6	0.062	44	41	<2
1609	8	2	2	2	1	>16	>16	32	6	0.125	42	41	<2
1669	8	2	2	2	1	>16	>16	32	12	0.062	40	38	<2

1670	8	2	2	2	1	>16	>16	32	10	0.125	40	36	<2
1675	16	2	4	4	1	>16	>16	4	27	0.125	43	36	<2
1676	>128	2	8	2	1	8	>16	4	26	0.25	32	16	<2
1681	>128	32	16	>16	>32	>16	>16	>64	6	>4	6	34	<2
1683	>128	>64	>32	>16	>32	>16	>16	>64	6	>4	6	6	<2
1685	>128	>64	>32	>16	>32	>16	>16	>64	6	>4	6	6	<2
1686	>128	>64	>32	>16	>32	>16	>16	>64	6	>4	6	6	<2
1687	16	2	4	2	1	>16	>16	4	20	>4	6	23	<2
1689	<4	2	2	2	0.5	8	4	2	24	0.125	34	30	<2
1690	<4	1	1	1	0.5	>16	4	2	23	2	29	30	<2
1701	64	32	16	>16	>32	>16	>16	64	6	>4	6	25	<2
1702	32	>64	16	>16	>32	>16	>16	>64	6	>4	6	26	<2
1703	32	16	8	>16	32	>16	>16	>64	6	>4	6	27	<2
1777	>128	>64	>32	>16	>32	>16	>16	>64	6	>4	6	6	<2
1778	>128	>64	>32	>16	>32	>16	>16	>64	6	>4	6	6	<2
1783	64	>64	16	>16	>32	>16	>16	>64	6	>4	6	21	<2
1784	>128	>64	>32	>16	>32	>16	>16	>64	6	>4	6	6	<2
1785	>128	>64	>32	>16	>32	>16	4	>64	6	>4	6	6	<2
1788	>128	>64	>32	>16	>32	>16	>16	>64	6	>4	6	6	<2
1803	32	16	8	8	1	8	>16	2	23	1	13	17	<2
1815	8	>64	>32	>16	>32	>16	8	>64	17	2	16	26	<2
1819	8	2	2	2	1	>16	4	4	20	>4	19	24	<2
1826	>128	>64	>32	>16	>32	>16	>16	>64	6	>4	6	6	<2
1837	32	8	8	>16	0.5	>16	>16	2	21	1	16	17	<2
1838	>128	>64	>32	>16	>32	>16	>16	>64	6	>4	6	6	<2
1839	8	2	2	2	0.5	>16	4	2	21	0.25	26	24	<2
1841	<4	2	2	1	2	>16	8	4	6	0.5	18	23	<2
1842	>128	>64	>32	>16	>32	>16	>16	>64	6	>4	6	6	<2
1847	16	2	8	2	1	>16	>16	16	16	1	16	23	<2
1868	>128	>64	>32	>16	>32	>16	>16	>64	6	>4	6	6	<2
1869	>128	32	>32	>16	>32	>16	>16	8	10	>4	6	12	<2
1871	32	2	8	2	0.5	2	1	4	20	1	17	18	<2
1878	64	16	8	8	1	>16	>16	4	23	0.5	20	13	<2
1879	32	16	8	8	1	>16	>16	2	21	0.5	18	15	<2
1884	64	>64	>32	>16	>32	>16	>16	>64	6	>4	6	6	<2
1885	>128	32	16	8	4	4	8	2	21	>4	12	17	<2
1894	8	2	2	2	0.5	>16	8	4	20	>4	10	20	<2
1939	8	2	2	2	1	2	>16	1	24	0.006	24	21	<2
1940	8	2	2	2	1	8	4	4	18	0.062	31	25	<2
1942	32	2	8	4	0.5	8	>16	2	21	0.5	18	17	<2
1943	8	2	4	2	1	>16	8	4	20	0.25	23	23	<2
1944	32	2	4	2	0.5	>16	>16	1	23	0.125	24	21	<2
1959	8	2	2	2	1	2	8	1	24	0.062	30	26	<2
1960	>128	>64	16	2	4	8	8	4	21	0.125	24	26	<2
1961	>128	>64	>32	>16	1	>16	>16	16	17	0.5	19	15	<2
1962	8	2	4	2	1	>16	8	4	20	0.5	24	25	<2
1964	8	2	2	2	1	2	<0.125	>64	6	0.125	26	25	<2
1985	8	2	2	2	1	2	8	1	25	0.06	26	23	<2
1986	64	16	8	8	1	>16	>16	2	20	>4	21	20	<2
1989	32	2	8	8	1	>16	>16	32	24	1	18	16	<2
1993	8	2	2	2	0.5	>16	>16	2	23	0.25	24	20	<2
1994	16	8	4	2	0.5	>16	>16	>64	6	0.5	21	26	<2
2008	>128	16	8	8	1	>16	>16	2	20	0.5	20	20	<2
2014	>128	>64	>32	>16	>32	>16	>16	>64	6	>4	6	6	<2
2023	>128	>64	>32	>16	>32	>16	>16	>64	6	>4	6	26	<2
2218	8	2	2	2	0.5	>16	4	2	22	0.06	33	30	<2
2224	32	>64	16	4	8	>16	>16	>64	6	>4	6	18	<2
2237	16	2	16	8	1	>16	>16	2	24	>4	12	25	<2
2238	>128	>64	>32	>16	>32	>16	>16	>64	6	>4	6	6	<2
2240	64	>64	>32	4	>32	>16	>16	16	12	>4	6	14	<2
2276	>128	16	16	8	1	>16	>16	32	6	>4	6	17	<2
2279	>128	32	16	8	4	>16	>16	>64	6	>4	6	15	<2

2280	>128	>64	>32	>16	>32	>16	>16	>64	6	>4	6	6	<2
2310	>128	>64	>32	>16	>32	>16	>16	>64	6	>4	6	6	<2
2355	>128	>64	>32	>16	>32	>16	>16	>64	6	>4	6	6	<2
2386	>128	>64	16	>16	>32	>16	>16	>64	6	>4	6	21	<2
2447	32	32	16	>16	>32	>16	>16	>64	6	>4	6	23	<2
2588	64	8	>32	>16	>32	>16	>16	>64	6	>4	6	16	<2
2590	16	2	8	2	1	>16	>16	4	23	0.5	16	18	<2
2594	>128	>64	>32	>16	>32	>16	>16	>64	6	>4	6	6	<2
2622	>128	>64	>32	>16	>32	>16	>16	>64	6	>4	6	6	<2
2623	>128	>64	>32	>16	>32	>16	>16	>64	6	>4	6	6	<2
2638	32	>64	>32	>16	>32	>16	>16	>64	6	>4	6	12	<2
2639	16	>64	>32	>16	>32	>16	>16	>64	6	>4	6	6	<2
2642	32	>64	>32	>16	>32	>16	>16	>64	6	>4	6	26	<2
2922	32	2	8	2	1	>16	>16	>64	6	>4	6	21	<2
2941	>128	>64	>32	>16	>32	>16	>16	>64	6	>4	6	6	<2
2943	16	2	8	4	1	>16	>16	4	23	0.5	21	20	<2
2946	>128	>64	>32	>16	>32	>16	>16	>64	6	>4	6	6	<2
2948	>128	>64	>32	>16	>32	4	4	>64	6	>4	6	6	<2
2954	>128	>64	>32	>16	>32	>16	>16	>64	6	>4	6	12	<2
2956	64	>64	>32	>16	>32	>16	>16	>64	6	>4	6	14	<2

TZP: piperacillin/tazobactam; CAZ: ceftazidime; FEP: cefepime; CZA: ceftazidime/avibactam; CT: ceftalozane/tazobactam; IPM: imipenem; MEM: meropenem; AMK: amikacin; TOB: tobramycin; CIP: ciprofloxacin; LVX: levofloxacin; ATM: aztreonam; CL: colistin

Table S5. Quality control parameters of the sequenced genomes.

Clinical Isolate	No. contigs	Size (bp)	GC (%)	N50	N90	L50	L90	Coverage (%)	Depth (x)
56	118	6837302	66.14	170165	56194	15	40	96.11	91.25
70	46	6294002	66.42	362902	83198	7	21	95.3	76.88
74	64	6084458	66.51	202402	49308	11	35	96.17	65.56
76	122	6829495	66.15	163436	56736	13	40	96.27	64.26
77	78	6701535	65.91	237761	71135	9	27	96.12	92.33
78	71	6704066	65.91	270742	81812	9	24	95.31	79.06
92	83	6697594	65.92	231958	58161	10	30	95.29	56.62
127	81	6698945	65.92	263195	63711	9	28	96.11	82.89
180	81	6710801	66.09	295112	71271	8	27	95.31	69.18
220	98	6943609	65.86	316897	57403	8	33	96.06	64.1
221	36	6187628	66.8	449091	173632	5	14	96.07	57.61
249	80	6719363	66.17	274195	67355	8	27	96.41	100.57
250	68	6729362	66.16	287357	85110	9	23	96.35	78.4
264	97	6949978	65.97	370215	71082	6	24	96.08	77.18
265	72	7078871	65.77	271547	90310	9	28	96.35	81.54
281	63	6837179	66.14	357342	82967	7	22	96.35	72.65
282	91	7240344	65.91	237255	69059	11	33	96.35	80.53
283	96	6964038	65.99	219563	65468	11	33	96.35	85.54

296	109	6926784	66.01	164590	51254	13	37	96.35	84.11
317	140	6978451	66.08	237107	62105	9	29	96.34	66.32
318	93	6980239	66.08	322171	75698	7	23	96.36	96.28
319	141	7357612	65.61	162276	41217	15	48	96.16	100.42
321	154	7346277	65.61	182251	41762	13	47	96.34	95.72
618	79	6817692	65.97	239168	91584	9	25	95.17	79.7
629	74	6827300	65.97	204180	91584	11	28	96.78	61.3
660	119	7278498	65.41	211916	60382	12	35	96.28	67.31
665	65	6880470	65.98	298062	90923	9	24	96.29	66.41
787	90	7030881	65.81	265706	75283	10	29	94.87	79.66
788	167	6955076	65.83	153749	42642	16	50	98.57	71.92
789	99	7057777	65.81	218292	68364	12	34	98.6	60.68
790	127	7084594	65.81	191032	45343	13	38	97.98	81.19
805	49	6305283	66.49	366527	88209	8	21	96.37	78.7
806	74	6392244	66.35	292064	80935	7	22	96.36	72.06
807	54	6351251	66.47	340754	84697	7	22	96.35	73.06
808	67	6967079	66	368801	90804	7	23	96.28	64.55
990	99	7044609	66.07	322173	77363	8	26	96.42	65.14
993	57	6820682	66.01	288083	90923	9	24	96.41	57.85
1038	80	6703216	65.91	241269	63711	10	29	96.28	74.04
1043	107	6846693	66.14	191610	52174	12	38	96.3	76.33
1060	56	6582268	66.12	277126	106234	8	23	95.3	71.91

1062	84	6922844	65.96	245040	71271	9	31	94.85	84.65
1063	70	6706691	65.91	333480	71144	6	21	96.1	73.88
1087	166	6994279	65.97	181250	41269	13	41	96.09	97.98
1189	112	6961230	66	181625	56411	12	37	96.27	60.28
1395	112	6950078	66.02	170732	51254	11	38	96.35	62.03
1489	65	6769444	66.02	224048	106229	9	25	96.35	77.48
1516	84	6811371	66.07	204962	69975	10	32	96.57	93.29
1545	44	6403263	66.41	538830	140179	5	14	96.57	86.6
1553	51	6729934	66.13	508101	133757	6	15	96.28	56.32
1573	64	6777913	66.02	294407	119939	7	22	96.54	60.45
1574	51	6721699	66.13	535888	100554	5	16	96.5	96.13
1590	55	6720232	66.12	370210	100554	7	20	92.81	73.52
1593	54	6722839	66.13	406873	94924	6	20	92.8	51.98
1597	43	6442612	66.39	428622	207960	5	13	96.37	59.95
1601	61	6731894	66.12	294224	106720	8	20	96.39	71.19
1609	61	6721397	66.12	365703	78784	6	20	96.7	74.24
1669	79	6756747	66.11	334723	92787	7	20	96.69	49.43
1670	60	6256769	66.51	389571	79761	7	18	95.97	72.39
1675	57	6272117	66.52	394926	89924	7	19	95.95	82.76
1676	92	6569828	66.23	321135	100013	8	20	96.3	86.03
1681	60	6779727	66.02	232250	88944	9	24	96.41	82.07
1683	143	6881464	66.15	234988	67747	10	32	96.35	79.78

1685	109	7324962	65.43	267518	67959	10	30	96.36	87.45
1686	63	6880071	65.96	336987	70811	8	21	82.75	23.68
1687	62	6690457	66.08	302782	98192	6	20	83.39	57.11
1689	442	6769875	65.66	61930	9058	28	134	96.4	95.48
1690	108	6806905	66	292774	71610	8	24	96.25	60.27
1701	85	7155402	65.72	294991	75957	10	26	96.87	60.17
1702	78	7118173	65.76	291439	82817	10	27	96.3	81.62
1703	79	7152653	65.72	271645	87807	9	26	96.08	55.81
1777	100	7020712	65.89	293039	70439	10	29	94.56	84.26
1778	116	7082953	65.79	242813	55367	10	31	95.89	71.68
1783	120	7073720	65.79	223518	51136	11	33	95.31	61.43
1784	109	6842343	66.21	377596	71891	5	24	95.28	38.19
1785	70	7008101	65.91	346644	74324	7	24	96.18	65.23
1788	70	6741356	66.14	226819	83326	10	27	96.57	52.04
1803	63	6738496	66.04	430332	81065	6	19	96.19	58.24
1815	61	6455165	66.34	400676	111604	6	18	83.6	75.15
1819	103	6897446	65.94	226648	68498	10	33	96.21	54.62
1826	54	6369688	66.44	332478	95287	6	18	97.44	70.95
1837	52	6375056	66.45	411319	103680	6	16	96.26	41.55
1838	52	6280094	66.54	472151	89746	4	13	96.27	75.13
1839	54	6281518	66.54	369636	219075	6	14	96.41	54.11
1841	88	6992874	65.87	304990	68376	7	27	96.4	59.58

1842	60	6410646	66.3	356562	91802	5	19	96.29	78.39
1847	103	6126898	66.26	122608	33154	14	51	95.32	70.58
1868	107	6844909	65.7	199041	47679	11	33	95.26	40.34
1869	103	6840331	65.7	211387	62478	11	32	96.4	74.36
1871	64	6319503	66.5	416438	71893	6	19	96.39	43.25
1878	58	6322260	66.5	359441	80439	7	20	95.29	77.87
1884	63	6425031	66.28	319735	86727	8	22	95.29	66.96
1885	73	6412781	66.29	267877	70655	9	26	96.3	48.88
1886	42	6383056	66.47	431218	108026	6	18	96.41	86.38
1894	42	6390380	66.46	339786	95423	7	21	96.33	81.32
1940	61	6287344	66.53	280045	94174	7	19	96.32	85.2
1942	47	6282113	66.51	620168	87673	5	14	96.29	54.04
1943	93	7013256	65.81	331263	71271	7	27	96.29	45
1944	85	7003822	65.81	299370	91696	8	25	96.27	46.18
1959	50	6354284	66.57	292967	75438	7	25	96.27	52.01
1960	64	6384608	66.51	542998	88454	5	15	96.31	67.17
1961	43	6317969	66.51	425438	141313	6	15	96.44	88.12
1962	78	6885755	66	294174	68498	8	26	96.31	87.78
1985	61	6347808	66.39	350024	106175	6	18	95.8	52.77
1986	52	6283827	66.53	359824	136626	7	17	95.84	67.39
1989	54	6567531	66.25	316245	76475	6	21	96.28	66.35
1993	103	6720911	66.05	307265	71271	8	25	96.21	84.57

1994	53	6393198	66.41	342500	94020	6	21	96.46	66.38
2008	123	6902555	66.07	163095	48111	13	41	96	15.99
2014	127	6943896	66.02	150052	48263	14	44	96.23	83.12
2023	52	6768297	66.11	349434	130707	7	20	96.29	57.93
2218	118	7065644	65.86	225921	70507	11	31	95.3	96.02
2224	116	7065490	65.86	242752	80574	11	30	96.28	53.17
2237	81	6586002	66.06	299222	74834	8	24	96.29	58.7
2238	204	7375293	65.38	154870	36878	17	59	96.31	30.4
2240	170	7389722	65.37	190627	38013	13	47	96.31	37.87
2276	110	7208788	65.74	160234	40108	13	43	95.27	47.37
2279	103	7209838	65.73	162210	52083	12	40	96.35	71.16
2280	111	6989813	66.11	258604	79777	8	26	96.39	85.7
2310	108	6957260	66.01	203335	52190	12	36	96.34	71.32
2355	126	6971170	65.99	155110	47546	14	44	96.1	73.68
2386	66	7048584	65.81	284563	87816	9	23	96.25	63.23
2447	101	7101313	65.76	186908	45343	13	38	96.37	31.58
2588	141	7094955	65.38	146534	39998	14	48	95.29	61.69
2590	53	6287957	66.48	367646	106179	6	16	96.11	73.37
2594	122	7271702	65.41	205158	55302	11	35	95.94	67.47
2622	120	6990169	65.88	346644	61644	8	27	95.6	68.53
2623	104	7034019	65.83	212229	74293	11	33	96.43	80.99
2628	146	6885165	66.14	201890	61931	11	35	96.09	56.18

2629	143	6875899	66.16	201890	61931	10	33	96.41	50.36
2642	113	6995704	65.91	231357	66525	10	35	96.09	76.3
2922	113	6944046	65.95	231461	68376	10	34	96.19	65.15
2941	69	7035376	65.83	331206	74284	8	23	96.07	88.25
2943	73	6341237	66.41	239050	68429	9	26	92.93	52.99
2946	393	6826507	65.81	27860	9112	73	241	96.09	88.79
2956	128	6863978	66.15	341525	67766	7	25	96.29	66.31

Table S6. Sequence types (STs) of the studied isolates.

Isolate	ST	Isolate	ST	Isolate	ST	Isolate	ST
56	235	808	2130	1702	111	1985	3024
70	612	990	2731	1703	111	1986	169
74	274	993	233	1777	309	1989	Novel
76	235	1038	1662	1778	111	1993	309
77	1662	1043	235	1783	111	1994	2865
78	1662	1060	233	1784	2731	2008	235
92	1662	1062	309	1785	309	2014	235
127	1662	1063	1662	1788	235	2023	233
180	309	1087	235	1803	309	2218	308
220	3205	1189	235	1815	Novel	2224	308
221	3560	1395	235	1819	309	2237	3959
249	235	1489	233	1826	569	2238	3045
250	235	1516	389	1837	569	2240	3045
264	309	1545	274	1838	776	2276	155
265	111	1553	309	1839	776	2279	155
281	309	1573	233	1841	309	2280	2731
282	Novel	1574	309	1842	Novel	2310	235
283	Novel	1590	309	1847	Novel	2355	235
296	235	1593	309	1868	111	2386	111
317	2731	1597	4010	1869	111	2447	111
318	2731	1601	309	1871	3519	2588	3045
319	2731	1609	309	1878	3519	2590	209
321	2731	1669	309	1884	2336	2594	3045
618	253	1670	675	1885	2336	2622	309
629	253	1675	675	1886	2683	2623	309
660	3045	1676	1601	1894	2683	2628	2731
665	233	1681	233	1940	169	2629	2731
787	111	1683	2731	1942	274	2642	309
788	1284	1685	309	1943	309	2922	309
789	111	1686	309	1944	309	2941	309
790	111	1687	244	1959	Novel	2943	252
805	3579	1689	244	1960	Novel	2946	309
806	1336	1690	244	1961	274	2948	309
807	3579	1701	111	1962	309	2956	2731

Table S7. Number and type of variants identified in the analyzed isolates.

Clinical isolate	Variant type				
	Complex	DEL	INS	MNV	SNV
56	4006	252	299	211	44859
70	1476	166	166	104	23425
74	1474	158	169	52	23255
76	4015	254	301	246	44975
77	1482	166	170	127	23626
78	1474	163	166	93	23565
92	1460	164	171	100	23559
127	1495	165	174	121	23630
180	3816	266	308	288	44713
220	1629	160	181	90	24326
221	47271	761	600	2836	143873
249	3976	251	306	357	45061
250	3991	252	310	312	45001
264	3862	263	309	257	44811
265	1954	165	206	158	25512
281	3830	271	322	334	45020
282	5212	304	366	286	53320
283	5185	311	368	361	53440
296	3995	251	304	256	44940
317	1597	186	186	104	24331
318	1641	189	189	89	24358
319	1557	175	176	77	24244
321	1604	176	181	74	24352
618	3992	263	302	296	46340
629	3957	264	310	350	46395
660	1639	186	189	132	24111
665	1750	166	173	138	25783

787	1951	163	205	122	25310
788	4155	277	307	272	46290
789	1961	166	205	159	25519
790	1938	161	201	108	25392
805	1580	170	172	142	23393
806	1469	154	168	104	22790
807	1613	174	171	139	23434
808	6999	386	359	426	66356
990	1633	188	187	114	24404
993	1778	168	175	133	25803
1038	1492	167	177	133	23640
1043	3988	255	304	334	45047
1060	1749	170	174	108	25774
1062	3845	260	304	262	44800
1063	1512	167	170	129	23671
1087	3971	251	305	330	45025
1189	4005	251	302	249	44908
1395	3995	250	303	283	44975
1489	1783	164	174	93	25703
1516	1533	173	175	98	23889
1545	1645	169	181	143	24572
1553	3832	263	309	302	44908
1573	1741	166	174	139	25788
1574	3795	263	307	319	44876
1590	3832	364	309	289	44888
1593	3829	265	306	311	44892
1597	1722	168	186	139	25614
1601	3804	266	308	321	44905
1609	3849	265	301	250	44823
1669	3815	268	309	329	44918
1670	1450	162	176	148	23237

1675	1455	160	176	140	23274
1676	3971	270	293	317	44998
1681	1707	161	169	113	25574
1683	1605	185	185	108	24373
1685	3819	261	309	288	44826
1686	3736	264	305	311	44046
1687	1256	156	175	98	21088
1689	1213	147	167	75	20548
1690	2946	173	182	162	25897
1701	1968	163	204	140	25519
1702	1965	162	207	135	25489
1703	1954	162	208	133	25453
1777	3846	265	312	254	44823
1778	1963	162	204	129	25467
1783	1932	162	201	111	25411
1784	1616	187	189	133	24387
1785	3797	268	310	353	44911
1788	3994	261	298	297	45039
1803	3752	259	309	326	44204
1815	1531	165	178	119	23690
1819	3838	267	307	270	44792
1826	1527	172	195	89	23597
1837	1508	173	191	118	23611
1838	1597	169	159	163	24055
1839	1610	168	159	134	24036
1841	3838	265	306	245	44752
1842	1759	197	193	142	26329
1847	1742	189	191	163	26204
1868	1923	159	194	130	24608
1869	1870	155	193	105	24496
1871	1934	188	192	136	27412

1878	1934	194	193	148	27420
1884	1544	160	190	127	23796
1885	1540	160	187	86	23750
1886	1829	182	180	137	26363
1894	1831	184	181	140	26394
1940	1786	165	186	126	24919
1942	1523	168	176	110	23497
1943	3811	257	308	319	44915
1944	3852	260	310	300	44921
1959	43242	625	495	1070	137069
1960	47110	733	584	2257	143286
1961	1536	170	174	134	23538
1962	3808	261	304	271	44731
1985	1851	186	198	112	26640
1986	1776	165	184	132	24915
1989	3937	272	290	228	45004
1993	3687	258	302	307	43819
1994	3913	241	282	346	45140
2008	3978	250	305	276	44944
2014	3972	248	299	173	44730
2023	1741	167	173	118	25762
2218	4048	262	311	249	45729
2224	4031	264	314	270	45716
2237	1341	164	180	105	22163
2238	1608	182	180	98	24019
2240	1646	184	186	136	24157
2276	1614	179	184	96	24057
2279	1623	187	181	113	24095
2280	1623	184	191	116	24369
2310	3991	252	302	285	44976
2355	3972	250	293	178	44725

2386	1977	164	208	117	25480
2447	1913	161	200	81	25318
2588	1598	186	183	98	23979
2590	1538	177	176	122	23490
2594	1637	187	193	141	24125
2622	3812	267	308	330	44873
2623	3830	265	304	236	44731
2628	1586	183	180	76	24285
2629	1578	183	178	88	24291
2642	3826	265	307	230	44720
2922	3815	261	312	276	44793
2941	3833	262	305	318	44868
2943	1839	178	195	135	26249
2946	2836	143	165	58	37261
2948	3817	263	310	319	44865
2956	1633	183	180	84	24381

DEL: Deletions; INS: Insertions; MNV: Multiple Nucleotide Variants; SNV: Single Nucleotide Variants.

Table S8. Isolates selected, kinetics, and gene expression analyses for growth.

Clinical Isolate	Phylogroup	ST	IPM	MEM	Carbapenemase	OprD	Efflux pumps
70	PAO1	612	S	S	✗	Complete	✓
74	PAO1	274	S	S	✗	Complete	✓
92	PAO1	1662	R	I	✓	Complete	✓
221	PA7	3560	S	S	✗	Complete	✓
281	PA14	309	S	S	✗	Complete	✓
318	PAO1	2731	R	R	✓	Complete	✓
787	PA14	111	R	R	✓	Partial	✓
990	PAO1	2731	R	R	✓	Complete	✓
1778	PAO1	111	R	R	✗	Complete	✓
1841	PA14	309	R	R	✓	Partial	✓
1871	PAO1	3519	S	S	✗	Complete	✓
1878	PAO1	3519	R	R	✗	Complete	✓
1940	PAO1	169	R	I	✗	Partial	✓
1942	PAO1	274	R	R	✗	Complete	✓
1959	PA7	Novel	S	R	✗	Partial	✓
1962	PA14	309	R	R	✗	Complete	✓
2218	PA14	308	R	I	✗	Partial	✓
2948	PA14	309	I	I	✗	Partial	✓

ST: sequence type; IPM: imipenem; MEM: meropenem; S: susceptible; R: resistant; I: intermediate; ✓: presence; ✗: absence.

Table S9. Specific growth rates (μ) of selected isolates under three conditions (no antibiotic, with imipenem, and with meropenem).

Clinical isolate	Specific growth rate (h^{-1})		
	**No antibiotic**	**With imipenem**	**With meropenem**
**70**	0.536133333	0.409433333	0.4752
**74**	0.615	0.736766667	0.4126
**92***	0.3118	0.333	0.276566667
**221**	0.273233333	0.242733333	0.110933333
**281**	1.0994	1.399333333	1.214133333
**318***	0.153833333	0.2042	0.224633333
**787***	0.300833333	0.5135	0.305966667
**990***	0.356466667	0.2413	0.251333333
**1778***	0.348	0.456766667	0.276666667
**1841**	0.1411	0.1834	0.6179
**1871***	0.708566667	0.4938	0.525133333
**1878**	0.369766667	0.2947	0.348066667
**1940**	0.5697	0.245333333	0.275466667
**1942**	0.544233333	0.178666667	0.314333333
**1959**	0.7061	0.4781	0.374133333
**1962**	0.367866667	0.368733333	0.1425
**2218**	0.550633333	0.2463	0.386733333
**2948***	0.276733333	0.5817	0.4299

*Isolates with carbapenemase-encoding genes

Table S10. Lag time of selected isolates under three conditions (no antibiotic, with imipenem, and with meropenem).

Clinical Isolate	Lag time (h)		
	No antibiotic	With imipenem	With meropenem
**70**	4	4	6
**74**	2	6	6
**92***	4	4	4
**221**	4	4	4
**281**	4	4	4
**318***	6	6	6
**787***	4	4	4
**990***	4	4	4
**1778***	6	6	4
**1841**	4	12	24
**1871***	4	4	4
**1878**	6	10	8
**1940**	4	15	10
**1942**	4	18	18
**1959**	4	8	6
**1962**	4	4	24
**2218**	4	18	22
**2948***	4	10	6

*Isolates with carbapenemase-encoding genes

Table S11. log_2FoldChange values and interpretation for the genes *oprD, mexA, mexC, mexE*, and *mexY* in selected isolates under no antibiotic conditions.

Clinical Isolate	log₂FoldChange (vs PAO1)									
	oprD		***mexA***		***mexC***		***mexE***		***mexY***	
**70**	-3.71561623	↓	4.77736092	↑	-4.13280169	↓	-4.26016045	↓	-1.093910217	↓
**74**	-3.1538353	↓	0.20821762	↔	-4.87830226	↓	-4.65340233	↓	-0.595111847	↔
**92**	-8.60172081	↓	1.83452606	↑	-2.74464862	↓	-2.97338867	↓	-4.823303223	↓
**221**	-7.67912483	↓	-0.6465683	↓	-5.9670016	↓	0.86230469	↔	0.010442734	↔
**281**	-25.7145433	↓	-10.4308729	↓	-14.2330415	↓	-13.1583719	↓	-8.17169857	↓
**318**	-10.5065575	↓	-0.82484245	↓	-9.85207812	↓	-2.44298172	↓	2.023485184	↑
**787**	-13.9439869	↓	0.30162811	↔	-6.14930217	↓	-5.73016548	↓	0.231103897	↔
**990**	-12.7286511	↓	-2.25992584	↓	-5.96962229	↓	-3.29986191	↓	1.824213028	↑
**1778**	-15.0386353	↓	2.44977951	↑	-0.93464915	↔	-3.76849747	↓	5.813581467	↑
**1841**	-10.903677	↓	-0.6751976	↔	-7.97065036	↓	-5.27957153	↓	2.168485641	↑
**1871**	-3.54179382	↓	5.77178955	↑	1.77012761	↑	-3.00298309	↓	0.672807694	↔
**1878**	-6.93589401	↓	3.37485313	↑	-0.75867907	↔	-5.7771759	↓	-0.144115448	↔
**1940**	-4.07949257	↓	-0.73848915	↔	5.15984853	↑	-1.27078247	↓	2.31439209	↑
**1942**	-0.43841362	↓	1.50174141	↑	-0.00820414	↔	-2.51605034	↓	-1.898233414	↓
**1959**	-5.55513763	↓	1.28511047	↑	1.9079984	↑	-0.53812599	↔	0.081727982	↔
**1962**	-9.57207108	↓	-6.79378891	↓	-4.66280619	↓	-4.25068665	↓	0.782844544	↔
**2218**	-5.34689903	↓	-4.06809425	↓	-2.68916766	↓	-3.57872009	↓	2.069051743	↑
**2948**	-15.89328	↓	-5.86594009	↓	0.65290769	↔	-2.4546814	↓	5.841890335	↑

↑: Overexpression; ↓: Underexpression; ↔: No change in expression

Table S12. log_2FoldChange values and interpretation for the genes *oprD, mexA, mexC, mexE*, and *mexY* in selected isolates exposed to imipenem.

Clinical Isolate	log₂FoldChange (vs intrastrain)									
	oprD		***mexA***		***mexC***		***mexE***		***mexY***	
**70**	-2.256097794	↓	-13.64475632	↓	2.094600677	↑	2.48840332	↑	1.933368683	↑
**74**	-0.285470963	↔	-0.219404221	↔	1.157125473	↑	2.880607605	↑	-0.323144913	↔
**92**	-2.718370438	↓	-2.728223801	↓	-2.06567955	↓	0.061306	↔	3.239421844	↑
**221**	-8.683279037	↓	-0.766607285	↔	-2.752151489	↓	-5.307613373	↓	-3.996192932	↔
**281**	9.214945793	↑	13.84749317	↑	9.781067848	↑	14.93542957	↑	11.9638586	↑
**318**	1.26546669	↑	2.402927399	↑	4.745029449	↑	0.76966095	↔	2.289970398	↑
**787**	-0.767354965	↓	-1.706817627	↓	0.173213959	↓	-0.957778931	↔	1.363603592	↑
**990**	1.137065887	↑	4.430028915	↑	3.840934753	↑	1.437707901	↑	0.772361755	↑
**1778**	1.535167694	↑	-1.096654892	↓	0.946489334	↔	0.692867279	↔	-1.676942825	↓
**1841**	-4.65180397	↓	3.320148468	↑	1.513835907	↑	3.782680893	↑	-0.012701035	↔
**1871**	-9.365066528	↓	-3.491872787	↓	-4.745014191	↑	-7.861440659	↓	3.144638062	↑
**1878**	1.663124084	↑	0.18469429	↔	3.207353592	↑	2.674833298	↑	1.508062363	↑
**1940**	-0.803155899	↓	-3.205049515	↓	-2.301193237	↑	-2.056409836	↓	-1.320962906	↓
**1942**	0.654981613	↔	0.779159546	↔	1.986692429	↑	1.852968216	↑	1.771621704	↑
**1959**	4.407709122	↑	0.285997391	↔	4.70970726	↑	5.638334274	↑	8.232328415	↑
**1962**	4.089994431	↑	6.069301605	↑	4.937587738	↑	1.179271698	↑	3.326639175	↑
**2218**	2.569217682	↑	3.045963287	↑	2.474841118	↑	4.746208191	↑	4.712173462	↑
**2948**	3.665594101	↑	3.816640854	↑	-0.019054413	↔	0.127407074	↔	1.877622604	↑

↑: Overexpression; ↓: Underexpression; ↔: No change in expression

Table S13. log_2FoldChange values and interpretation for the genes *oprD, mexA, mexC, mexE*, and *mexY* in selected isolates exposed to meropenem.

Clinical Isolate	log₂FoldChange (vs intrastrain)									
	oprD		***mexA***		***mexC***		***mexE***		***mexY***	
**70**	-5.577363968	↓	-7.395519257	↓	-1.239995956	↓	-1.604436874	↓	-2.062871933	↓
**74**	-0.7144927979	↓	1.25834465	↑	0.9544582367	↔	-2.061513901	↓	0.4740753174	↔
**92**	3.014120102	↑	2.107702255	↑	1.236595154	↑	1.820667267	↑	4.372432709	↑
**221**	11.05755711	↑	-1.387878418	↓	-7.690395355	↓	-5.781259537	↓	-7.556991577	↓
**281**	9.978692055	↑	0.1767911911	↔	7.286257744	↑	9.047400475	↑	10.67952061	↑
**318**	-0.9551944733	↓	3.760206223	↑	5.987829208	↑	1.866464615	↑	-2.295154572	↓
**787**	-4.96585083	↓	-0.1596794128	↔	2.007102966	↑	-1.7554245	↓	0.9441776276	↔
**990**	0.8927879333	↓	0.4716644287	↔	1.482383728	↑	-0.2845783234	↔	-0.9378433228	↔
**1778**	1.521312714	↑	-3.087915421	↓	-1.390216827	↓	-1.532007217	↓	-3.284749985	↓
**1841**	-6.975728989	↓	-0.002833366394	↔	-2.296253204	↓	-0.3155632019	↔	-4.14667511	↓
**1871**	-0.9713687897	↓	-2.816387177	↓	0.3679542542	↔	-0.4425945282	↔	-1.093618393	↓
**1878**	4.412214279	↑	0.01373672485	↔	3.48654747	↑	5.366752625	↑	-1.417993546	↓
**1940**	-1.379398346	↓	0.2207584381	↔	-2.686578751	↓	0.7698707581	↔	0.1351928711	↔
**1942**	-1.022880554	↓	-3.189210892	↓	-0.7982158661	↔	-2.092666626	↓	2.997198105	↑
**1959**	8.049646378	↑	23.46814537	↑	3.81517081	↑	1.095373154	↑	7.500471115	↑
**1962**	1.907766342	↑	5.101701736	↑	2.003190994	↑	2.292444229	↑	1.659858704	↑
**2218**	-0.1018695831	↓	2.275075912	↑	-0.9090480804	↔	0.02667617798	↔	-1.793931961	↓
**2948**	11.62501144	↑	4.345504761	↑	1.20614624	↑	3.584957123	↑	0.7941379547	↔

↑: Overexpression; ↓: Underexpression; ↔: No change in expression

Table S14. log2FoldChange values and type of sequence variaton in efflux pump regulators genes.

Isolate	mexA_noAB	mexA_IPM	mexA_MEM	*mexR*	*nalC*	*nalD*
70	4.78	-13.64	-7.40	none	missense	none
74	0.21	-0.22	1.26	none	missense	synonymous
92	1.83	-2.73	2.11	synonymous	missense	synonymous
221	-0.65	-0.77	-1.39	synonymous	none	none
281	-10.43	13.85	0.18	synonymous	none	none
318	-0.82	2.40	3.76	synonymous	missense	missense
787	0.30	-1.71	-0.16	synonymous	missense	synonymous
990	-2.26	4.43	0.47	synonymous	missense	missense
1778	2.45	-1.10	-3.09	synonymous	missense	synonymous
1841	-0.68	3.32	0.00	synonymous	none	none
1871	5.77	-3.49	-2.82	synonymous	missense	none
1878	3.37	0.18	0.01	synonymous	missense	none
1940	-0.74	-3.21	0.22	missense	missense	none
1942	1.50	0.78	-3.19	none	missense	synonymous
1959	1.29	0.29	23.47	synonymous	none	none
1962	-6.79	6.07	5.10	synonymous	none	none
2218	-4.07	3.05	2.28	synonymous	none	none
2948	-5.87	3.82	4.35	synonymous	none	none

Isolate	mexC_noAB	mexC_IPM	mexC_MEM	*nfxB*
70	-4.13	2.09	-1.24	synonymous
74	-4.88	1.16	0.95	synonymous
92	-2.74	-2.07	1.24	synonymous
221	-5.97	-2.75	-7.69	missense
281	-14.23	9.78	7.29	missense
318	-9.85	4.75	5.99	synonymous
787	-6.15	0.17	2.01	synonymous
990	-5.97	3.84	1.48	synonymous
1778	-0.93	0.95	-1.39	synonymous
1841	-7.97	1.51	-2.30	missense
1871	1.77	-4.75	0.37	synonymous
1878	-0.76	3.21	3.49	synonymous
1940	5.16	-2.30	-2.69	synonymous
1942	-0.01	1.99	-0.80	synonymous
1959	1.91	4.71	3.82	missense
1962	-4.66	4.94	2.00	missense
2218	-2.69	2.47	-0.91	missense
2948	0.65	-0.02	1.21	missense

Isolate	mexE_noAB	mexE_IPM	mexE_MEM	*mexS*	*mexT*
70	-4.26	2.49	-1.60	missense	frameshift
74	-4.65	2.88	-2.06	missense	frameshift
92	-2.97	0.06	1.82	missense	frameshift
221	0.86	-5.31	-5.78	none	missense
281	-13.16	14.94	9.05	none	synonymous
318	-2.44	0.77	1.87	missense	frameshift
787	-5.73	-0.96	-1.76	missense	frameshift
990	-3.30	1.44	-0.28	missense	frameshift
1778	-3.77	0.69	-1.53	missense	frameshift
1841	-5.28	3.78	-0.32	none	synonymous
1871	-3.00	-7.86	-0.44	missense	frameshift
1878	-5.78	2.67	5.37	synonymous	frameshift
1940	-1.27	-2.06	0.77	missense	frameshift
1942	-2.52	1.85	-2.09	missense	frameshift
1959	-0.54	5.64	1.10	none	missense
1962	-4.25	1.18	2.29	none	synonymous
2218	-3.58	4.75	0.03	none	synonymous
2948	-2.45	0.13	3.58	none	synonymous

Isolate	mexY_noAB	mexY_IPM	mexY_MEM	*mexZ*
70	-1.09	1.93	-2.06	synonymous
74	-0.60	-0.32	0.47	synonymous
92	-4.82	3.24	4.37	synonymous
221	0.01	-4.00	-7.56	none
281	-8.17	11.96	10.68	none
318	2.02	2.29	-2.30	missense
787	0.23	1.36	0.94	synonymous
990	1.82	0.77	-0.94	missense
1778	5.81	-1.68	-3.28	synonymous
1841	2.17	-0.01	-4.15	none
1871	0.67	3.14	-1.09	synonymous
1878	-0.14	1.51	-1.42	synonymous
1940	2.31	-1.32	0.14	synonymous
1942	-1.90	1.77	3.00	synonymous
1959	0.08	8.23	7.50	none
1962	0.78	3.33	1.66	none
2218	2.07	4.71	-1.79	none
2948	5.84	1.88	0.79	none
